# Modelling the monthly abundance of *Culicoides* biting midges in nine European countries using Random Forests machine learning

**DOI:** 10.1186/s13071-020-04053-x

**Published:** 2020-04-15

**Authors:** Ana Carolina Cuéllar, Lene Jung Kjær, Andreas Baum, Anders Stockmarr, Henrik Skovgard, Søren Achim Nielsen, Mats Gunnar Andersson, Anders Lindström, Jan Chirico, Renke Lühken, Sonja Steinke, Ellen Kiel, Jörn Gethmann, Franz J. Conraths, Magdalena Larska, Marcin Smreczak, Anna Orłowska, Inger Hamnes, Ståle Sviland, Petter Hopp, Katharina Brugger, Franz Rubel, Thomas Balenghien, Claire Garros, Ignace Rakotoarivony, Xavier Allène, Jonathan Lhoir, David Chavernac, Jean-Claude Delécolle, Bruno Mathieu, Delphine Delécolle, Marie-Laure Setier-Rio, Bethsabée Scheid, Miguel Ángel Miranda Chueca, Carlos Barceló, Javier Lucientes, Rosa Estrada, Alexander Mathis, Roger Venail, Wesley Tack, Rene Bødker

**Affiliations:** 1 Division for Diagnostics and Scientific Advice, National Veterinary Institute, Technical University of Denmark (DTU), Lyngby, Denmark; 2 Department of Applied Mathematics and Computer Science, Technical University of Denmark (DTU), Lyngby, Denmark; 3 Department of Agroecology - Entomology and Plant Pathology, Aarhus University, Aarhus, Denmark; 4 Department of Science and Environment, Roskilde University, Roskilde, Denmark; 5 National Veterinary Institute (SVA), Uppsala, Sweden; 6 Faculty of Mathematics, Informatics and Natural Sciences, Universität Hamburg, Hamburg, Germany; 7 Bernhard Nocht Institute for Tropical Medicine, Hamburg, Germany; 8 Department of Biology and Environmental Sciences, Carl von Ossietzky University, Oldenburg, Germany; 9 Institute of Epidemiology, Friedrich-Loeffler-Institut, Greifswald, Germany; 10 Department of Virology, National Veterinary Research Institute, Pulawy, Poland; 11 Norwegian Veterinary Institute, Oslo, Norway; 12 Unit of Veterinary Public Health and Epidemiology, University of Veterinary Medicine, Vienna, Austria; 13 CIRAD, UMR ASTRE, 34398 Montpellier, France; 14 IAV Hassan II, Unité MIMC, 10 100 Rabat-Instituts, Morocco; 15 Institute of Parasitology and Tropical Pathology of Strasbourg, UR7292, Université de Strasbourg, Strasbourg, France; 16 EID Méditerranée, Montpellier, France; 17 Applied Zoology and Animal Conservation Research Group, University of the Balearic Islands, Palma, Spain; 18 Department of Animal Pathology, University of Zaragoza, Zaragoza, Spain; 19 Institute of Parasitology, National Centre for Vector Entomology, Vetsuisse FacultyInstitute of Parasitology, National Centre for Vector Entomology, Vetsuisse Faculty, University of Zürich, Zürich, Switzerland; 20 Avia-GIS NV, Zoersel, Belgium; 21 Meise Botanic Garden, Meise, Belgium

**Keywords:** *Culicoides* abundance, Random Forest machine learning, Spatial predictions, Europe, Environmental variables, *Culicoides* seasonality

## Abstract

**Background:**

*Culicoides* biting midges transmit viruses resulting in disease in ruminants and equids such as bluetongue, Schmallenberg disease and African horse sickness. In the past decades, these diseases have led to important economic losses for farmers in Europe. Vector abundance is a key factor in determining the risk of vector-borne disease spread and it is, therefore, important to predict the abundance of *Culicoides* species involved in the transmission of these pathogens. The objectives of this study were to model and map the monthly abundances of *Culicoides* in Europe.

**Methods:**

We obtained entomological data from 904 farms in nine European countries (Spain, France, Germany, Switzerland, Austria, Poland, Denmark, Sweden and Norway) from 2007 to 2013. Using environmental and climatic predictors from satellite imagery and the machine learning technique Random Forests, we predicted the monthly average abundance at a 1 km^2^ resolution. We used independent test sets for validation and to assess model performance.

**Results:**

The predictive power of the resulting models varied according to month and the *Culicoides* species/ensembles predicted. Model performance was lower for winter months. Performance was higher for the Obsoletus ensemble, followed by the Pulicaris ensemble, while the model for *Culicoides imicola* showed a poor performance. Distribution and abundance patterns corresponded well with the known distributions in Europe. The Random Forests model approach was able to distinguish differences in abundance between countries but was not able to predict vector abundance at individual farm level.

**Conclusions:**

The models and maps presented here represent an initial attempt to capture large scale geographical and temporal variations in *Culicoides* abundance. The models are a first step towards producing abundance inputs for R_0_ modelling of *Culicoides*-borne infections at a continental scale.
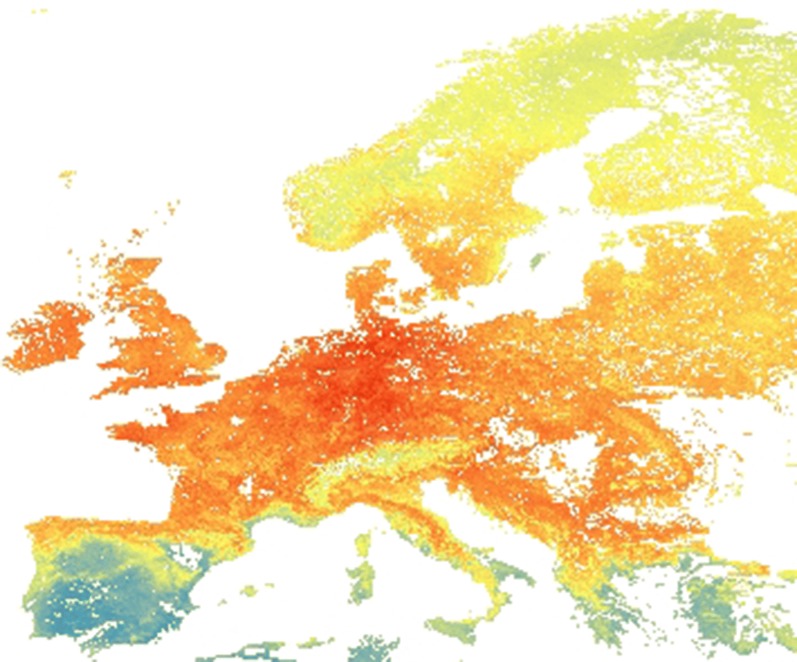

## Background

Biting midges of the genus *Culicoides* (Diptera: Ceratopogonidae) are small blood-sucking flies responsible for the transmission of viruses of veterinary importance in ruminants and equids. These viruses include bluetongue virus (BTV), Schmallenberg virus, and epizootic haemorrhagic disease virus or African horse sickness in equids [1]. In Europe, outbreaks of bluetongue and Schmallenberg have caused large economic losses to the livestock industry during recent decades [2, 3]. Bluetongue (BT) was previously restricted to the Mediterranean basin but started to spread in northern Europe in 2006 [1, 4, 5]. To prevent the virus from spreading further, the European Union initiated extensive entomological surveillance programmes in order to determine the *Culicoides* species composition and monitor their seasonal dynamics [6], and to determine vector-free periods for animal trade [7]. Several *Culicoides* studies have shown that BTV was transmitted in northern Europe by autochthonous Palaearctic *Culicoides* species [8] such as *C. obsoletus* (Meigen), *C. scoticus* Downes & Kettle [5, 9], *C. dewulfi* Goetghebuer [10] and *C. chiopterus* (Meigen) [11, 12].

Geographical and temporal variation in vector abundance are key determinants of the potential transmission of vector-borne diseases [13]. The potential disease transmission can be calculated as a R_0_ value, expressing the number of new cases generated from a single case when a pathogen is introduced into a naïve population [13, 14]. R_0_ estimates allow health authorities and decision makers to determine when and where possible disease outbreaks might occur. Hence, a series of actions to prevent further spread of the disease can be planned. Using entomological data collected on farms and environmental variables obtained from satellite imagery, it is possible to model and map the abundance of vectors. *Culicoides* abundance maps for Europe can be found either at a national [15–17] or a continental scale for *C. imicola* [18, 19] and for the Obsoletus ensemble [20]. The *Culicoides* maps available at a continental scale for Europe are usually created with abundance data collected within a limited area of the mapped region. The response is extrapolated after predicting beyond the domain of the sampled farms [18, 19, 21]. Therefore, there is still a need to produce data-validated European prediction maps across a wider range of unsampled regions for the main *Culicoides* vectors.

Machine learning techniques are algorithms that, like classical statistical models, can be used to predict an outcome using predictor variables. The machine learning technique Random Forests (RF) has been proven to outperform classical approaches for species distribution modelling such as generalized linear models (GLM) and logistic regression (LR) [22–24]. We hypothesised that *Culicoides* abundance may be predicted for a large area of Europe using a RF approach and climatic and environmental predictors. These predictors have proven effective in previous *Culicoides* studies [15, 23, 25, 26]. The entomological dataset covers nine countries and represents the largest entomological dataset aggregated to date comprising 595 sampled livestock farms with 30,626 trap collections and 8,539,420 recorded specimens. This extraordinary dataset has been used in a previous study to: (i) determine geographical variation in the start of the vector season at a continental scale for Europe; (ii) map the observed abundance by means of simple interpolation (no predictors used); and (iii) to analyse the seasonality of these vectors [27]. Additionally, in a second study, this dataset was used to map the probability of presence at a continental scale, introducing a method to reclassify those maps into classes (present, absent and uncertain) so they can be used for targeted surveillance and for decision making by veterinarian authorities. Results showed that it was possible to predict the probability of the monthly presence of host-seeking *Culicoides* females with a fair accuracy (AUC range: 0.92–0.97), especially for the southerly distributed *C. imicola* [28]. In this study, we used the *Culicoides* dataset to predict the geographical variation in the monthly vector abundance through nine European countries. We present average abundance maps per month for Obsoletus and Pulicaris ensembles (i.e. *C. pulicaris* (Linnaeus) and *C. punctatus* (Meigen)) and for *C. imicola*. We evaluated the predictive value of the maps and furthermore compared the resulting RF maps to maps created by interpolating the observed abundance. This was done to determine if the model including environmental predictors gave better predictions compared to simple interpolation.

## Methods

### *Culicoides* dataset

**Fig. 1:**
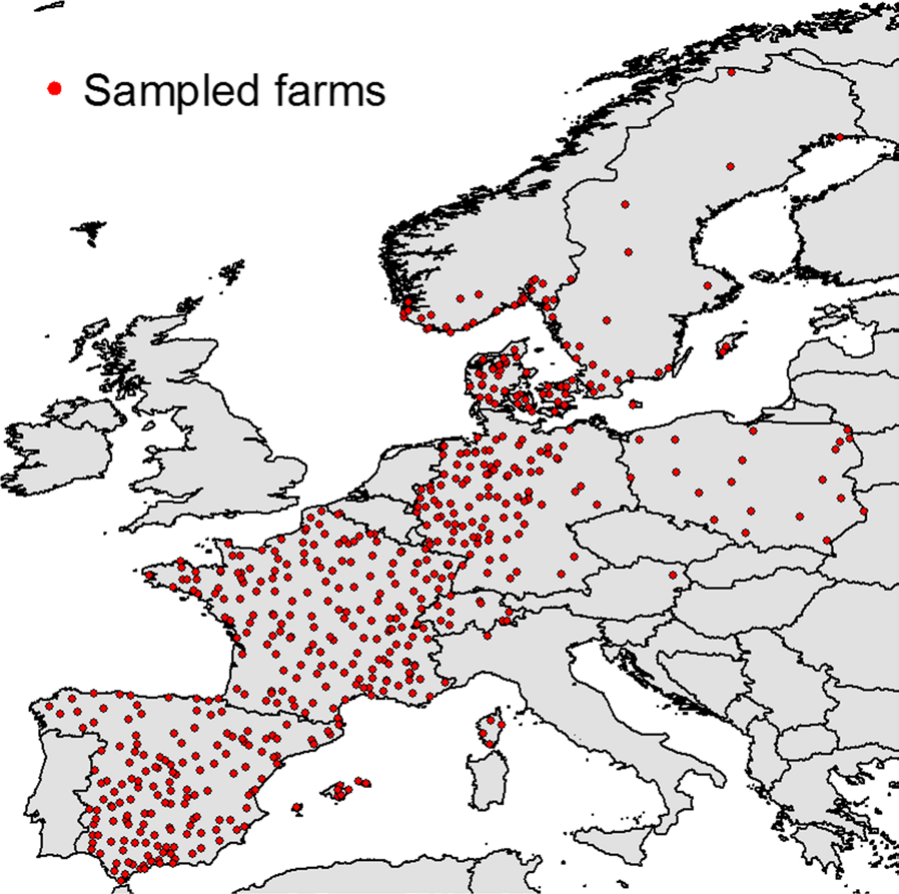
Entomological data from sampled farms in Europe during entomological surveys from 2007 to 2013 were used. Original data contained more farms but for this analysis, temporary traps at Danish farms were removed from the analysis

*Culicoides* data were collected from cattle, sheep and horse farms in Spain, France, Germany, Austria, Switzerland, Denmark, Norway, Sweden and Poland from 2007 to 2013. This same dataset was used in two previous studies [27, 28], but here, the Danish data from farms where traps were only operated for one night are removed. From a total of 350 farms sampled in Denmark, we only used 49 sentinel farms for the analysis. We did this to avoid the pseudo-replication of environmental conditions which would be created by the very high sampling density in Denmark compared to the rest of the study area (Fig. 1).

The dataset contained data from 595 sampled livestock farms with 30,626 trap collections and 8,539,420 specimens of *Culicoides* caught. Details on the collection of this dataset, sampling protocol and conversion factors can be found in [27] and therefore, we here only provide a summary. Black-light suction traps were placed outside each farm and were usually operational once a week during the sampling period from dusk to dawn. Specimens were identified by morphology to species level for *C. imicola* and aggregated when they belonged to the Obsoletus or Pulicaris ensembles. We here use the term “ensemble” to refer to a group of sympatric species for which morphological identification is sometimes difficult or not possible during routine surveillance, and without phylogenetic meaning [27, 28].

The dataset was divided into 12 independent monthly subsets according to the month of catch. For each monthly dataset, we first calculated the mean abundance at each farm for each year sampled and then log-transformed the mean abundance using the formula log_10_ (mean abundance +1). This resulted in 12 monthly datasets where each farm contained as many records as number of years sampled. These abundance estimates were treated as independent observations, despite originating from the same farm.

### Predictor variables

We used environmental and climatic data together with estimates of production animal density, land cover features and soil types as predictor variables of biting midge abundance. All predictors were in raster format and they were pre-processed and resampled to fit a resolution of 1 × 1 km pixel size. We resampled and pre-processed the raster layers using R software (version 3.6.1) [29] (package *raster*) [30].

Environmental predictors were derived from a MODIS temporal series from 2001 to 2012. We examined mid-infrared (MIR), daytime land surface temperature (dLST), night-time land surface temperature (nLST), enhanced vegetation index (EVI) and normalised difference vegetation index (NDVI), and each variable had been processed using a Temporal Fourier Analysis (TFA) [31] (Table 1).

**Table 1:** Environmental and land cover predictors used to model *Culicoides* abundance

Source	Code	Description
Modis (Fourier-transformed) (2001–2012)	MIR	Mid-infrared
	dLST	Daytime land surface temperature
	nLST	Night-time land surface temperature
	NDVI	Normalised difference vegetation index
	EVI	Enhanced vegetation index
Bioclim^b^ (1960–1990)	BIO 1	Annual mean temperature
	BIO 2	Mean diurnal range: mean of monthly (max. temp - min. temp)
	BIO 3	Isothermality (BIO 2/BIO 7) (×100)
	BIO 4^a^	Temperature seasonality (standard deviation × 100)
	BIO 5^a^	Max. temperature of warmest month
	BIO 6^a^	Min. temperature of coldest month
	BIO 7	Temperature annual range (BIO 5 – BIO 6)
	BIO 8	Mean temperature of wettest quarter
	BIO 9^a^	Mean temperature of driest quarter
	BIO 10^a^	Mean temperature of warmest quarter
	BIO 11^a^	Mean temperature of coldest quarter
	BIO 12^a^	Annual precipitation
	BIO 13	Precipitation of wettest month
	BIO 14	Precipitation of driest month
	BIO 15	Precipitation seasonality (coefficient of variation)
	BIO 16^a^	Precipitation of wettest quarter
	BIO 17^a^	Precipitation of driest quarter
	BIO 18	Precipitation of warmest quarter
	BIO 19	Precipitation of coldest quarter
	Altitude	Digital elevation model (DEM)
Corine Land Cover^c^	CLC 12	Non-irrigated arable land
	CLC 13	Permanently irrigated land
	CLC 18	Pastures
	CLC 19	Annual crops associated with permanent crops
	CLC 20	Complex cultivation patterns
	CLC 21	Land principally occupied by agriculture with significant areas of natural vegetation
	CLC 22	Agro-forestry areas
	CLC 23	Broad-leaved forest
	CLC 24	Coniferous forest
	CLC 25	Mixed forest
	CLC 26	Natural grasslands
	CLC 29	Transitional woodland-shrub
	CLC 35	Inland marshes
	CLC 40	Water courses
	CLC 41	Water bodies

^a^Variables discarded during pre-processing analysis due to high correlation

^b^https://www.worldclim.org/

^c^https://land.copernicus.eu/pan-european/corine-land-cover/clc-2012

The Bioclim raster dataset (version 1.4) was obtained from the Worldclim online database [32]. Animal density data for cattle, goats and sheep were obtained from FAO “GeoNetwork” [33] (Table 1).

We used CORINE land cover classification map [34] at a resolution of 250 m, extracting 16 classes that we considered relevant to *Culicoides* occurrence (Table 1). Each class was transformed into binary images according to the presence or absence of the class. From these binary images, we calculated the number of pixels that contained the class for every 1 km^2^ and created maps displaying the frequency of each class per pixel. These raster files were used as individual predictors.

We identified pairs of highly correlated variables and removed one of the variables from each correlated pair from the analysis. In total, 25 predictors were removed in order to optimize the processing time: BIO 4, BIO 5, BIO 6, BIO 10, BIO 11, BIO 12, BIO 16, BIO 17, BIO 9, MIRMiN, MIRMaX, dLSTMiN, dLSTMaX, nLSTMiN, nLSTMaX, NDVIMiN, NDVIMaX, EVIMiN, EVIMaX, MIRVR, dLSTVR, nLSTVR, NDVIVR, EVIVR, dLSTD3 and nLSTD3.

The same set of predictors were used previously to model the probability of presence of *Culicoides* in Europe [27]. Table 1 summarizes the variables used as predictors.

Additionally, in this study we included “soil types” among the predictor variables. This is a raster file showing the principal soil types [35]. “Soil types” were added as a single predictor variable with the different soil types as factors. This raster layer contains 10 classes showing the main soil types in Europe and has been previously used as an independent variable for predicting tick abundance in Scandinavia [36].

As the monthly mean abundance of *Culicoides* showed some variation over the years (Additional file 1: Figure S1), we decided to include the year of sampling as a predictor variable in each monthly model. We added the variable “year” as a set of seven binary dummy variables (one for each year) and generated a prediction map for each year.

For each month, we used the seven annual prediction maps to calculate: (i) the average predictions over the seven years; and (ii) the coefficient of variation as: CV = standard deviation/mean. These calculations were made for each pixel using the values corresponding to each year (*n* = 7).

We considered this average map to be the best prediction of abundance in a future year. A standard deviation map was previously created to show the variability in predictions made for *C. impunctatus* in Scotland [26]. Instead we chose to calculate the coefficient of variation. The coefficient of variation calculates the variation based on “mean units” and allows for comparison of variation in samples with different means.

### Modelling approach

We used the machine learning method Random Forests (RF) [37] to predict the abundance of biting midges. A RF consists of an ensemble of decision trees (a forest) in which each tree contributes with a prediction for a given observation. The overall prediction for that observation is the average of all individual tree’s predictions in the forest [38]. The RF technique has previously been used to model the geographical distribution and/or abundance of vectors such as mosquitoes [23], biting midges [17, 39] and parasites (*Fasciola hepatica*) [40]. The advantages of decision trees include their robustness against outliers and their capability to identify complex interactions, including non-linear relationships between the response and predictor variables. Additionally, a RF ranks the predictors with respect to importance. This is typically done by calculating the improvement in the prediction error when each variable is permuted [21, 38]. We used R 3.4.1 [29] (packages *caret* [41], *randomForest* [42] and *raster* [30]) to model and predict abundance data using the above-mentioned raster files as predictors. The *caret* package looks for the best number of candidate variables for splitting the data at each node (m_try_) using a tuning grid. In this study, the m_try_ parameter was set to 30 and the number of trees was set to 750 (ntree = 750). We used five-fold cross-validation for the tuning process.

### Validation

We divided each monthly dataset into a training and test set at random. The training set included 70% of the total farms sampled that month, and the test set included the remaining 30% of the farms. For each month, we used the training set to train a RF model. The resulting model was then used to predict the abundance of each observation belonging to the test set (external validation) [26, 40]. To analyse model performance, we plotted the predicted values as a function of the observed values for all test set observations. We used the normalised root mean square error (nRMSE = RMSE/mean of predicted values) of the test set in order to compare results from different months. Lower nRMSE values indicated better model performance. Additionally, we used QQ-plots to evaluate the normality of the residuals to determine model performance.

### Interpolation model

We decided to compare the predictions obtained by RF modelling to simple spatial interpolation, a method that does not requires any predictors. To compare these two different approaches, we calculated the monthly average per farm, using the previous abundance averages calculated per year and ran new RF models. We used the same dataset to geographically interpolate the average abundance. Thus, we obtained two abundance maps per month. We used the interpolation algorithm inverse distance weighted (IDW) which was used to predict the abundance for this dataset in a previous analysis [27]. We used the IDW function (Geostatistical Analyst Tool) in ArcMap 10.1 (ESRI, Redlands, CA, USA) with the following settings: power equal to 2; minimum neighbours equal to 10; and maximum neighbours equal to 15.

To validate both models, we applied the external validation method, using 70% of the data as the training set and 30% of the data as a test set and calculated the residuals (observed minus predicted values in the test dataset). We evaluated the model performance by plotting the predicted values against the observed values and comparing the nRMSE.

## Results

### Model performance

In general, the nRMSE for each month showed that RF performed fairly well for the Obsoletus ensemble (nRMSE range: 0.38–2.01) and less well for the Pulicaris ensemble (nRMSE range: 0.65–12.97) but poorly for *C. imicola* (nRMSE range: 1.47–12.27). For the three ensembles/species, the performance of the RF models varied across months, with nRMSE values higher than 1 during the colder months (Table 2). Months with a nRMSE higher than 2 indicate that the predicted site abundances in that month differ from the observed sites abundances by at least 100 individuals (on average), and thus should be interpreted with caution, as nRMSE of these magnitudes indicate low predictive power.

**Table 2:** Normalised root mean square error (nRMSE), in units of log_10_ abundance, calculated for each month and each *Culicoides* ensemble/species

Month	Obsoletus ensemble	Pulicaris ensemble	*C. imicola*
	nRMSE	nRMSE	nRMSE
January	2.01	12.97	**1.36**
February	1.78	3.21	1.97
March	1.06	3.29	1.60
April	0.48	**0.63**	2.92
May	0.86	1.58	2.73
June	0.56	0.84	2.25
July	**0.38**	0.65	2.95
August	0.60	0.94	2.53
September	0.65	0.85	1.49
October	1.38	1.05	1.47
November	0.84	1.34	1.74
December	1.34	2.07	2.19

*Note*: Bold values show the lowest nRMSE

**Fig. 2:**
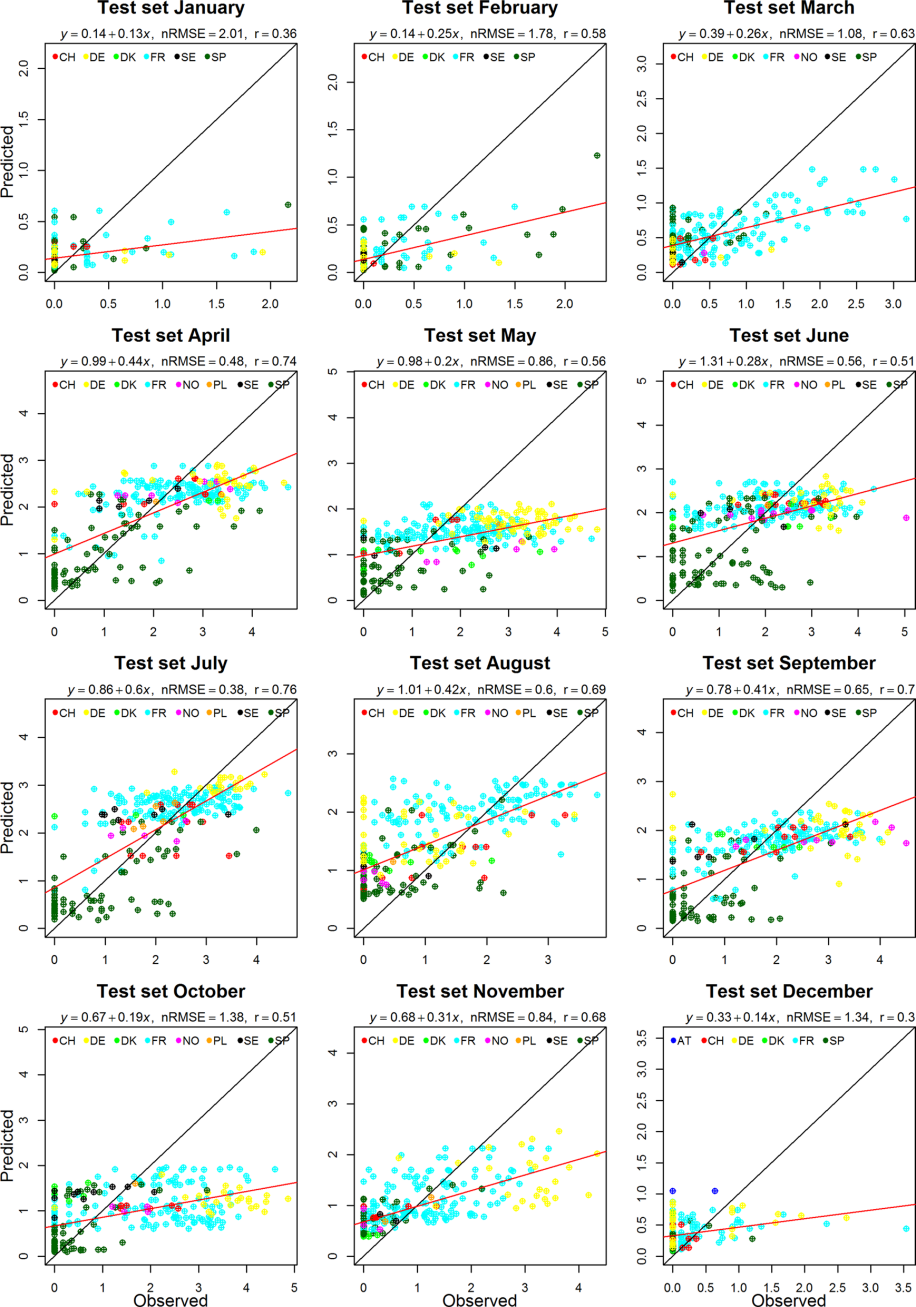
Scatter plot of the predicted and observed abundance of the Obsoletus ensemble. Red line: best linear model fit; black line: perfect model fit. Note that scales depict log_10_-values and varies across different months. For all months, *P* < 0.05. *Abbreviations*: AT, Austria; CH, Switzerland; DE, Germany; DK, Denmark; FR, France; PL, Poland; SE, Sweden; SP, Spain; NO, Norway

For the Obsoletus ensemble in general, there was a positive linear correlation between predicted and observed abundances (Fig. 2). The best model was for July with an nRMSE of 0.38, followed by April with a nRMSE of 0.48. The model was weakest for January, where the scatterplot of observed *versus* predicted values showed a cloud with a weak linear trend and an nRMSE of 2.01 (Fig. 2). The highest predicted abundances were observed for Germany (May-November) followed by France, while the lowest predicted abundances were found for Spain. We found a large variation in the predictions from farms with observed null abundance for all months, but this variation decreased as the observed abundance increased (Fig. 2). For January, February, March, November and December (winter period), the QQ plots showed the residuals were not normally distributed, nevertheless the rest of the year the QQ plots showed that the residuals were normally distributed, indicating a good model performance (Additional file 1: Figure S2).

**Fig. 3:**
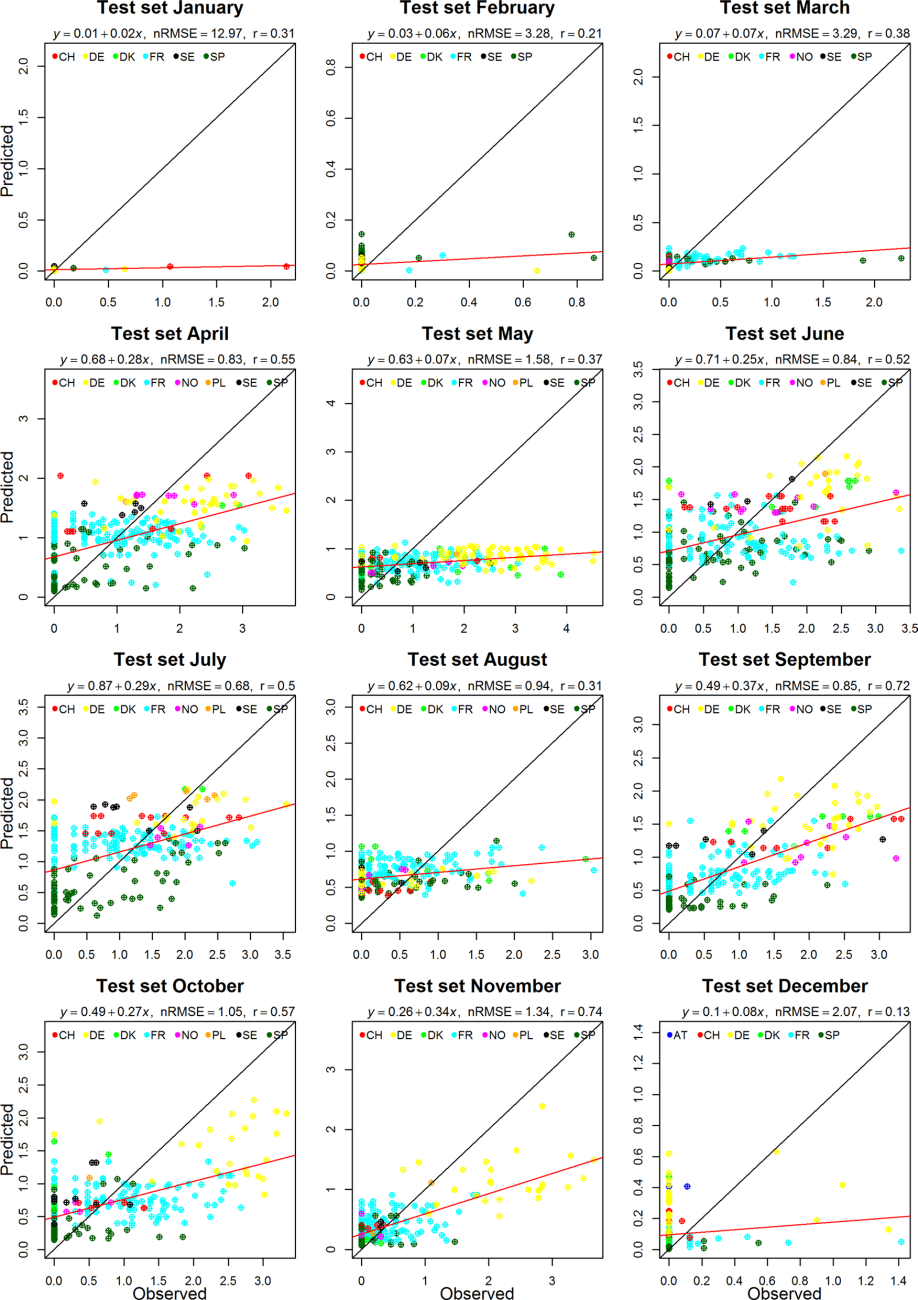
Scatter plot of the predicted and observed abundance of the Pulicaris ensemble. Red line: best linear model fit; black line: perfect model fit. Note that scales depict log_10_-values and varies across different months. For all months, *P* < 0.05. *Abbreviations*: AT, Austria; CH, Switzerland; DE, Germany; DK, Denmark; FR, France; PL, Poland; SE, Sweden; SP, Spain; NO, Norway

Performance of the Pulicaris ensemble model was poorer than for the Obsoletus ensemble model, resulting in a minimum nRMSE of 0.65 in July (Table 2). Nevertheless, a positive relationship was observed between the predictions and the observed abundance (Fig. 3). QQ plots of the residuals showed that the models for January, February, March, August, September, November and December were not normally distributed, indicating low model performance. For the remaining months, the models performed better as the QQ plot showed normally distributed residuals (Additional file 1: Figure S3).

Performance of the *C. imicola* models was poor, as shown by the high nRMSE values obtained for all months. The minimum nRMSE was found for October with a value of 1.47. The monthly models were incapable of predicting the high observed abundance of *C. imicola*, resulting in similar low predictions throughout the range of observed abundances. For January, all observed abundance values were zero and thus, it was not possible to fit a regression line (Table 2). The residuals were not normally distributed for any month (data not shown).

### Average abundance of annual maps

**Fig. 4:**
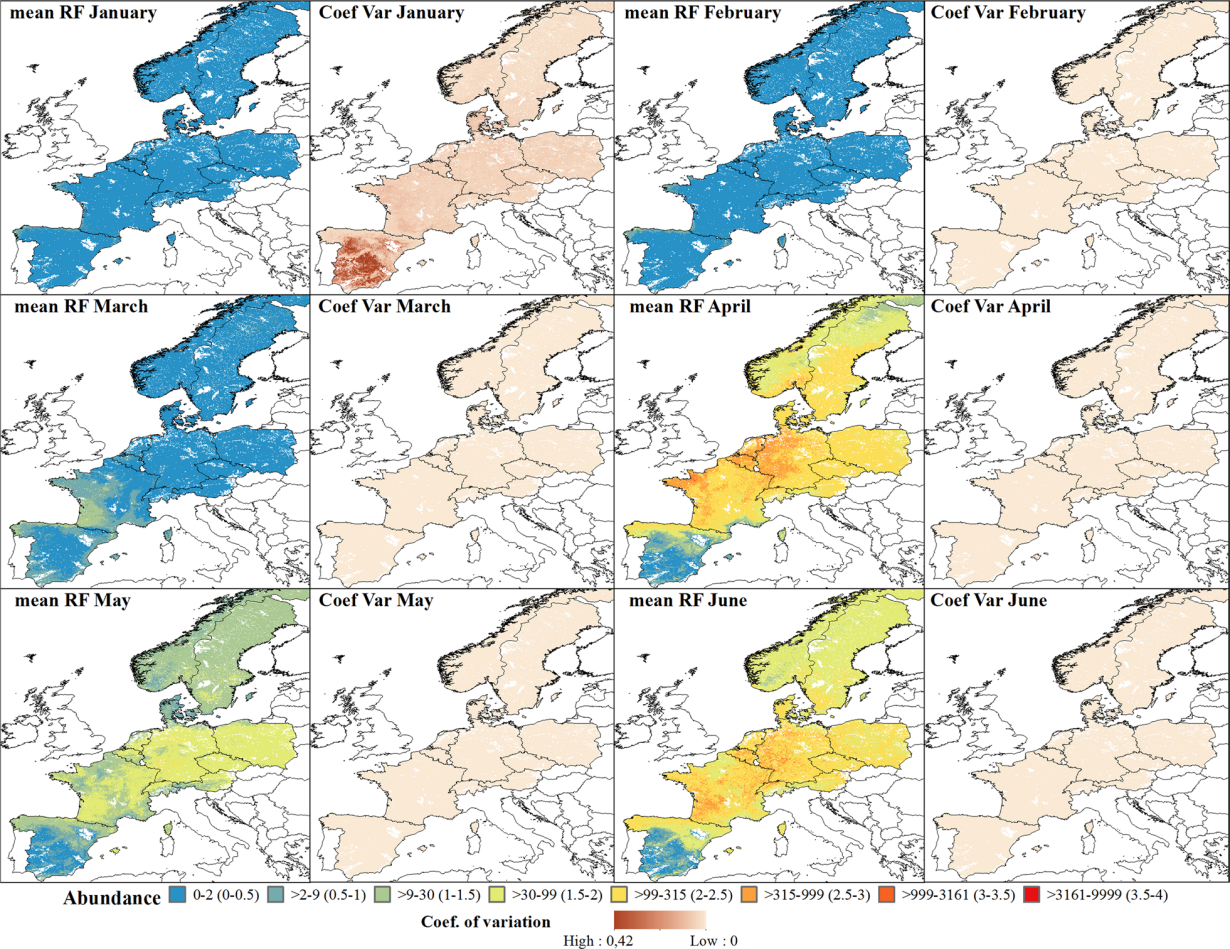
Predicted abundance maps from January to June for the Obsoletus ensemble. The mean predictions were calculated per pixel using the seven prediction maps made for each year. Values are shown on a log_10_ scale. Coefficient of variation maps highlight the areas with a larger variation in predictions over the seven-year study period

**Fig. 5:**
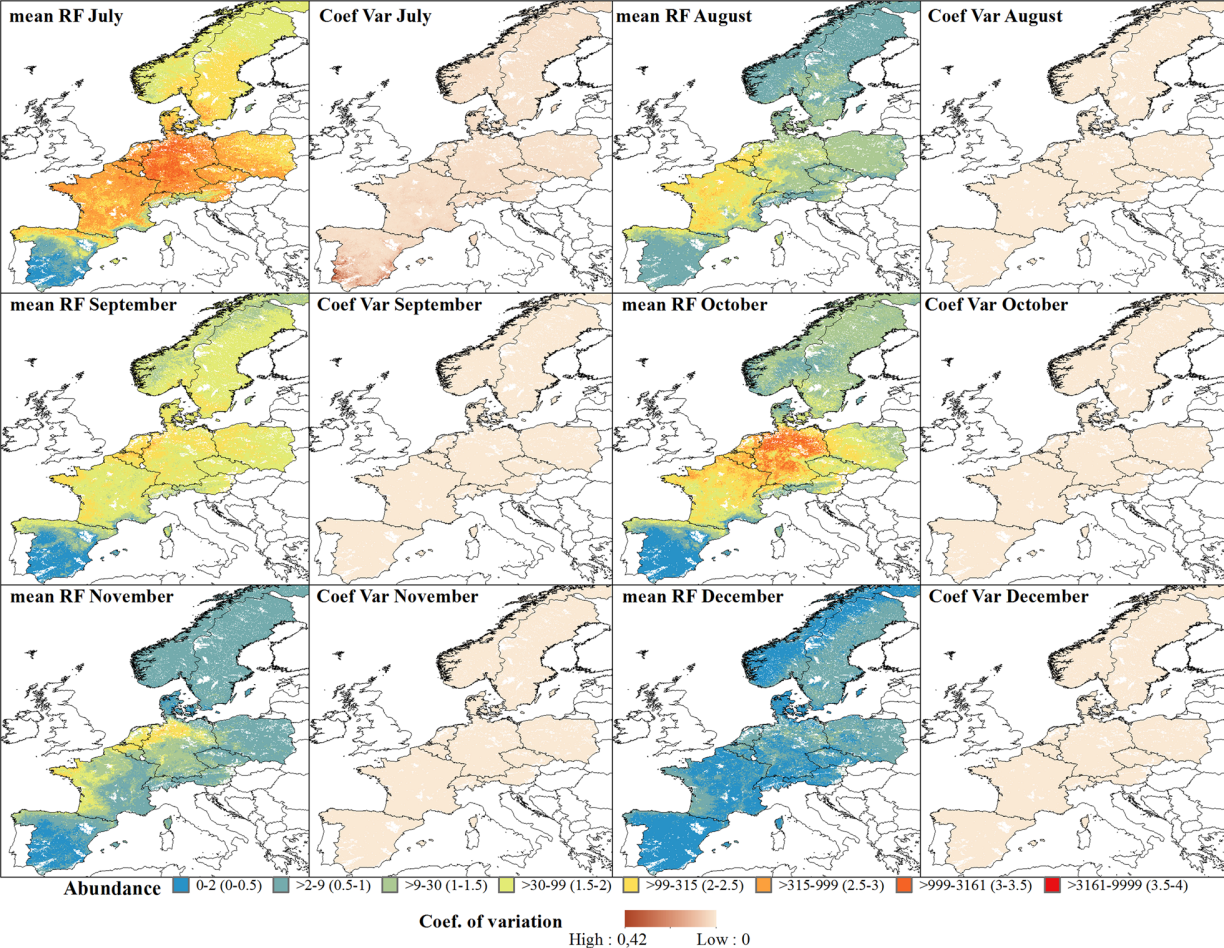
Predicted abundance maps from July to December for the Obsoletus ensemble. The mean predictions were calculated per 1 km^2^ pixel using the seven prediction maps made for each year. Values are shown on a log_10_ scale. Coefficient of variation maps highlight areas with large variation in predictions over the seven-year study period

The predicted abundance for the Obsoletus ensemble showed a seasonal pattern with high abundance during the summer months and low abundance during the winter months. In March, the predicted abundance of the Obsoletus ensemble started to increase in western France and along the north coast of Spain (Fig. 4). From April onwards, abundance increased gradually over the entire study area, reaching approximately 10,000 individuals per night in July in Germany (Fig. 5). Abundance decreased in August but increased again in September and October to approximately 10,000 *Culicoides* per night in Germany, although inter-annual variation also increased for October. After this, abundance decreased in November, with the areas of highest abundance located in Germany. From December to February, abundance was predicted to be very low (< 10 specimens or null) (Fig. 5). The coefficient of variation maps showed that the highest coefficient of variation between years was found in Spain, indicating that this area had the highest variation in predictions across all the years (Figs. 4, 5).

**Fig. 6:**
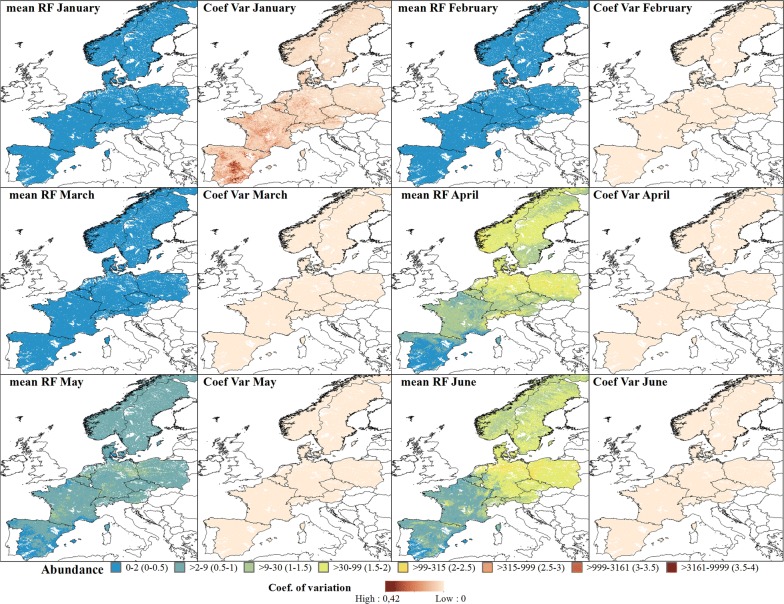
Predicted abundance maps from January to June for the Pulicaris ensemble. The mean predictions were calculated per pixel using the seven prediction maps made for each year. Values are shown on a log_10_ scale. Coefficient of variation maps highlight the areas with a larger variation in predictions over the seven-year study period

**Fig. 7:**
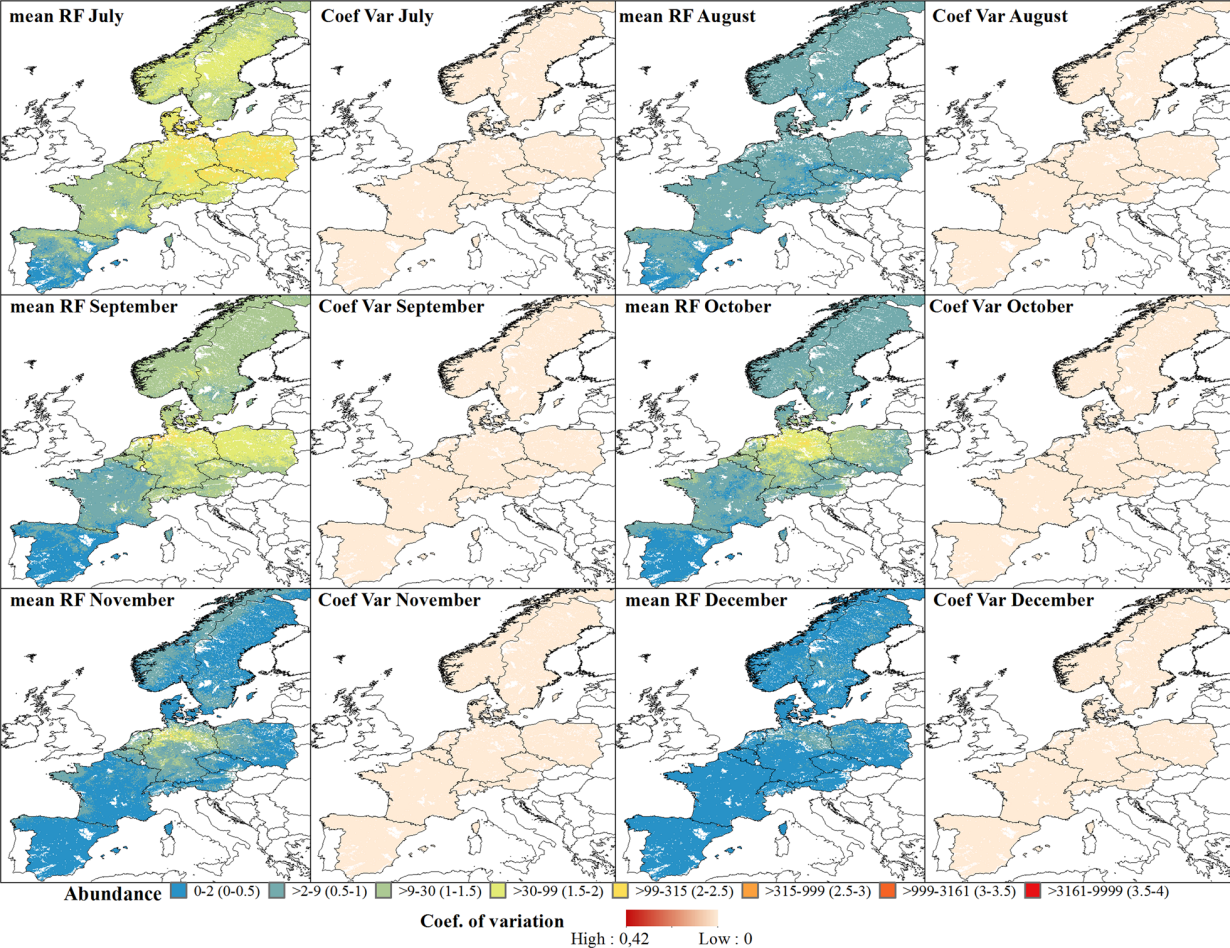
Predicted abundance maps from July to December for the Pulicaris ensemble. The mean predictions were calculated per pixel 1 km^2^ using the seven prediction maps made for each year. Values are shown on a log_10_ scale. Coefficient of variation maps highlight areas with large variation in predictions over the seven-year study period

The predicted abundance for the Pulicaris ensemble showed a similar seasonal pattern with an increase in abundance from April (Fig. 6), a decrease in May, followed by higher abundances in June, with a peak of approximately 1000 individuals per 24 h. From April, the highest abundance was predicted in northern Germany, with a decreasing abundance towards western France and medium abundance towards Poland. This pattern was maintained until October (except in August where there was a decrease in the abundance), and abundance started to decrease gradually in November, with northern Germany again having the highest abundance (Fig. 7). In general, the Pulicaris ensemble showed a more easterly distribution (Germany, Poland and Scandinavia) and a much lower overall abundance compared to the Obsoletus ensemble.

**Fig. 8:**
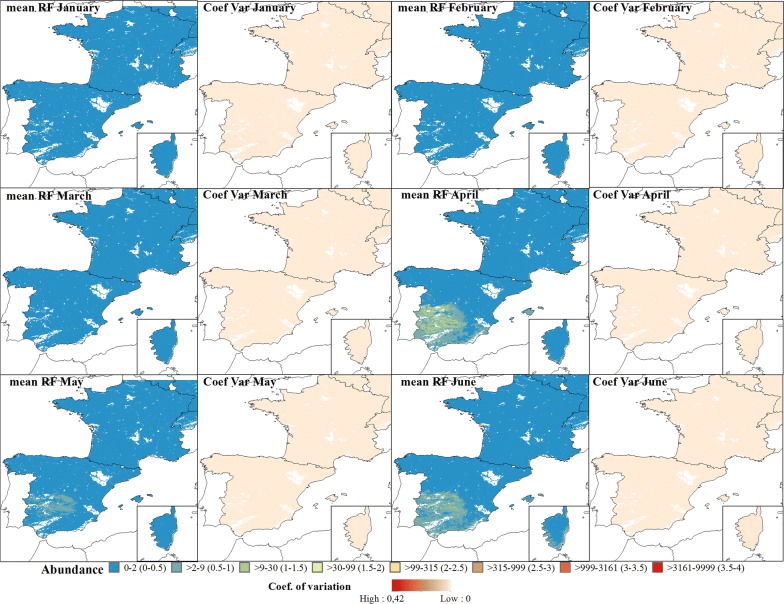
Predicted abundance maps of the Iberian Peninsula and Corsica (bottom right corner) from January to June for *C. imicola*. The mean predictions were calculated per pixel using the seven prediction maps made for each year. Values are shown on a log_10_ scale. Coefficient of variation maps highlight the areas with a larger variation in predictions over the seven-year study period

**Fig. 9:**
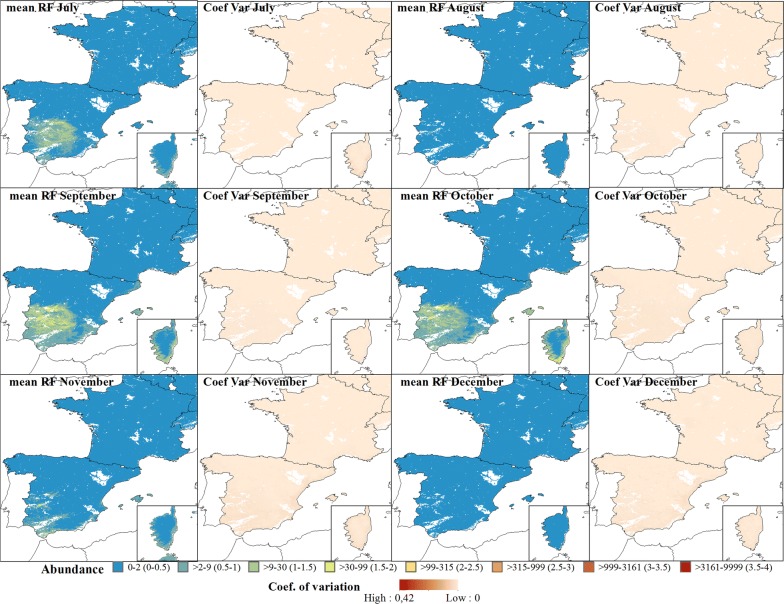
Predicted abundance maps of the Iberian Peninsula and Corsica (bottom right corner) from July to December for *C. imicola.* The mean predictions were calculated per 1 km^2^ pixel using the seven prediction maps made for each year. Values are shown on a log10 scale. Coefficient of variation maps highlight areas with large variation in predictions over the seven-year study period

*Culicoides imicola* was predicted to have very low abundance in January and February (< 10 individuals), with the abundance increasing gradually throughout March, until it peaked in July and October in central Spain and on the coast of Corsica (Figs. 8, 9).

### Variable importance

The five most important predictor variables identified for each month and for each *Culicoides* group are reported in Table 3. In general, considering only the months with a nRMSE ≤ 2, the most important variables for the Obsoletus ensemble were related to temperature (LST) and precipitation (BIO 18). For the Pulicaris ensemble, the most important variables were related to temperature. For *C. imicola*, the most important variables were related to precipitation and temperature.

**Table 3:** The five most important variables given by the Random Forests (RF) models for each month

Month	Ensemble or species	Variable 1	Variable 2	Variable 3	Variable 4	Variable 5
January	Obsoletus	NDVI A2 (100)	BIO 13 (99.29)	dLST A0 (94.14)	MIRP3 (91.60)	dLST A1 (83.34)
	Pulicaris	dLST DA (100)	BIO 18 (98.32)	dLST A0 (97.28)	BIO 14 (95.91)	MIR A1 (94.91)
	*C. imicola*	NDVI A0 (100)	dLST A1 (95.94)	year.2012 (92.02)	dLST DA (88.44)	EVI A1 (84.53)
February	Obsoletus	dLST A1 (100)	BIO 2 (89.01)	Altitude (79.35)	BIO 3 (78.19)	BIO 7 (76.32)
	Pulicaris	rec_snow (100)	NDVI A3 (91.51)	BIO 1 (88.21)	BIO 18 (85.53)	BIO 3 (84.6)
	*C. imicola*	MIRDA (100)	BIO 18 (96.60)	EVI P1 (94.60)	MIR D3 (92.40)	nLST P3 (92.06)
March	Obsoletus	BIO 8 (100)	dLST A1 (89.42)	BIO 1 (60.88)	nLST A0 (58.24)	Altitude (47.88)
	Pulicaris	dLST A1 (100)	BIO 1 (97.29)	MIRD1 (97.24)	Altitude (96.54)	dLST P1 (95.80)
	*C. imicola*	BIO 1 (100)	MIRP2 (91.79)	BIO 18 (91.49)	BIO 14 (86.05)	BIO 15 (84.94)
April	Obsoletus	EVI A1 (100)	BIO 18 (57.19)	BIO 14 (98.44)	dLST P2 (95.81)	EVI P1 (94.00)
	Pulicaris	dLST A0 (100)	BIO 18 (77.71)	BIO 1 (73.82)	dLST P2 (67.63)	BIO 14 (66.36)
	*C. imicola*	dLST P2 (100)	BIO 1 (99.74)	BIO 15 (93.88)	MIR P1 (93.46)	MIR D1 (93.21)
May	Obsoletus	BIO 3 (100)	BIO 18 (66.19)	BIO 8 (48.85)	BIO 2 (44.25)	nLST P1 (33.82)
	Pulicaris	year.2010 (100)	nLST P3 (53.54)	BIO 15 (32.67)	dLST P3 (31.48)	BIO 1 (30.68)
	*C. imicola*	nLST A0 (100)	BIO 14 (91.81)	BIO 1 (89.24)	BIO 7 (83.80)	year.2008 (79.33)
June	Obsoletus	BIO 18 (100)	dLST P1 (62.27)	nLST P1 (57.27)	MIR A0 (55.11)	BIO 2 (48.97)
	Pulicaris	BIO 1 (100)	Goat (55.07)	year.2008 (48.19)	BIO 8 (46.77)	nLST P3 (42.38)
	*C. imicola*	year.2008 (79.33)	year.2008 (74.75)	dLST P2 (74.30)	BIO 7 (65.81)	BIO 18 (48.15)
July	Obsoletus	BIO 18 (100)	BIO 2 (69.01)	BIO 14 (68.50)	Altitude (63.25)	nLST A2 (59.09)
	Pulicaris	BIO 1 (100)	BIO 18 (85.69)	EVI P3 (82.03)	dLST P3 (80.52)	dLST A0 (75.92)
	*C. imicola*	BIO 14 (100)	year.2008 (74.75)	dLST P2 (74.30)	BIO 7 (65.81)	BIO 18 (48.15)
August	Obsoletus	nLST A2 (100)	nLST A2 (90.07)	BIO 1 (87.11)	nLST A0 (83.53)	year.2008 (66.38)
	Pulicaris	year.2011 (100)	year.2012 (49.76)	nLST A2 (49.56)	nLST A0 (48.00)	nLST P2 (37.90)
	*C. imicola*	BIO 1 (100)	BIO 18 (97.20)	MIRD1 (96.48)	dLST P2 (96.20)	EVI P1 (95.47)
September	Obsoletus	BIO 18 (100)	year.2012 (62.45)	nLST P1 (45.62)	nLST A2 (44.68)	MIR P2 (43.26)
	Pulicaris	nLST A2 (100)	BIO 1 (86.34)	dLST P2 (80.35)	dLST A0 (76.10)	BIO 8 (71.45)
	*C. imicola*	BIO 1 (100)	nLST A0 (90.67)	BIO 14 (83.13)	dLST P2 (78.91)	BIO 18 (70.83)
October	Obsoletus	BIO 3 (100)	BIO 18 (38.70)	year.2012 (32.36)	nLST A2 (23.50)	BIO 2 (23.18)
	Pulicaris	nLST A2 (100)	year.2012 (64.97)	BIO 1 (41.18)	BIO 3 (40.17)	nLST P2 (38.33)
	*C. imicola*	BIO 14 (100)	BIO 1 (95.56)	nLST A0 (91.46)	BIO 13 (81.09)	BIO 15 (73.79)
November	Obsoletus	nLST A2 (100)	BIO 3 (93.01)	EVI A0 (62.95)	year.2011 (58.13)	nLST P3 (53.28)
	Pulicaris	BIO 8 (100)	nLST A2 (93.51)	Altitude (87.21)	dLST P1 (82.30)	dLST P2 (77.51)
	*C. imicola*	BIO 14 (100)	BIO 13 (96.53)	BIO 1 (91.16)	nLST A0 (72.20)	nLST P1 (67.13)
December	Obsoletus	Altitude (100)	NDVI A0 (97.44)	dLST A1 (97.30)	EVI A2 (92.83)	EVI D2 (92.24)
	Pulicaris	nLST A2 (100)	nLST P3 (97.92)	dLST A0 (92.55)	Altitude (92.51)	dLST P2 (91.47)
	*C. imicola*	Goat (100)	year.2008 (76.26)	year.2011 (75.42)	BIO 15 (63.56)	EVI DA (61.48)

*Notes*: Numbers in parentheses indicate the importance of the variables. The top most important variables (“Variable 1” column) have a value of 100

### Comparison between interpolation and RF performance

The RF maps seemed to be smoother than the interpolation maps (Additional file 1: Figures S4a, b; S5a, b; S6a, b). This is because the interpolation maps showed higher predicted values in the surroundings of the farms used for training. However, when zooming in on the maps it becomes apparent that the interpolation models resulted in a smooth transition from farm to farm, while the predictions from the environment-driven RF actually varied pixel by pixel (Additional file 1: Figure S7).

When comparing the mean nRMSE through all the months for the three *Culicoides* taxa/species, the RF model performed only slightly better than the interpolation (Table 4).

**Table 4:** Normalized RMSE values (nRMSE) for the RF models and interpolation for January to December

Month	Obsoletus ensemble		Pulicaris ensemble		*C. imicola*	
	nRMSERF	nRMSE Interpolation	nRMSE RF	nRMSE Interpolation	nRMSE RF	nRMSE Interpolation
January	2.03	3.22	14.70	17.57	1.36	4.35
February	1.92	1.81	3.76	4.54	2.16	3.16
March	1.17	1.25	3.59	3.51	1.70	2.70
April	0.46	0.53	0.8	0.76	2.73	3.66
May	0.56	0.51	0.89	0.81	2.64	5.34
June	0.62	0.50	0.78	0.66	2.03	2.69
July	0.38	0.38	0.71	0.65	2.44	3.01
August	0.71	0.68	1.45	1.30	2.38	2.81
September	0.57	0.55	0.86	0.75	1.53	2.12
October	0.65	0.50	0.88	0.72	1.56	2.69
November	0.76	0.67	1.33	1.15	1.75	2.18
December	1.51	1.65	2.13	2.59	3.20	3.82
Total mean	0.94	1.02	2.65	2.91	2.12	3.21

*Notes*: RF and interpolation were performed using the average abundance. The mean for all months and for each method is shown in the last row

The scatterplots for the predicted and observed values for both the interpolation and the RF models were generally quite similar but the interpolation models predicted a higher abundance compared to the RF models. The range predicted by the interpolation method was closer to the observed range than the more limited range predicted by the RF method, eventhough the interpolation predictions were not more precise than RF predictions (i.e. they were no closer to the best fitted line) (Additional file 1: Figures S8, S9, S10).

## Discussion

We modelled the abundance of the Obsoletus and Pulicaris ensembles as well as *C. imicola* using the machine learning technique Random Forests (RF), and predicted the vector abundance on a continental scale using entomological data obtained from national monitoring and research programmes in nine European countries. We used catch data from 31,429 *Culicoides* traps over the years 2007–2013. The model prediction differed according to the months and especially in the winter period, when the predictive power was low. The predicted abundance maps presented here were based on the largest entomological dataset generated to date for *Culicoides.* There is a great need for *Culicoides* abundance data, e.g. for R_0_ modelling in Europe. The resulting maps show major geographical abundance patterns and give some insight into seasonal dynamics on a monthly scale. Although large datasets were available, the predictions produced here are associated with large uncertainties, and the models were not able to capture the observed large variation in abundance on a local scale.

RF performance for predicting abundance varied with season. In general, the error (nRMSE) was higher during the winter months, possibly because fewer farms were sampled during the winter or because *Culicoides* are known to use indoor refugia [43] that might lead to poorer correlations between ambient climatic conditions and abundance. The poorer performance of the RF models for some months may be explained by limitations caused by using a dataset merged from different sources or by limitations related to the RF algorithm. In the RF algorithm, predictions for extreme observations (low or high abundance found within farms) were computed by averaging the training dataset outcomes in the terminal nodes and as a result, large values will necessarily be underestimated and low values overestimated [41]. Another reason for poor performance may be that the remote sensing predictors used here were not the key drivers (or not the only key drivers) of *Culicoides* abundance on European farms. It may be that landscape conditions on a finer scale, such as farm practice, management and microhabitats are more important drivers of vector abundance on the farms. The poor performance of our models may also be due to the resolution of the predictors; 1 km^2^ may not be the optimal resolution for capturing certain local landscape features that could affect the local abundance at the farm level, like soil moisture conditions that determines the presence of small breeding sites. For example, *C. imicola* oviposits on mud or semi-moist areas, at the margin of ponds or close to leaking irrigation pipes [44, 45].

We observed differences between the maps obtained using RF and interpolation according to scale; on a local scale, the interpolation method resulted in generally smooth surface maps (Additional file 1: Figure S7). As expected, RF produced maps showing more patchy variation in abundance than models created through interpolation by distance. RF models did not perform dramatically better than simple interpolation methods, suggesting that the available land cover classes had limited predictive power for *Culicoides* abundance. The lack of importance of land cover in predicting vector abundance is also supported by the RF decision trees mainly selecting climate variables over land cover variables as predictors. Since the interpolation predictions were not much worse compared to RF (which included land cover predictors), we conclude that large-scale variation in *Culicoides* abundance across Europe can be explained by drivers with a gradual change. Climate, and especially temperature, has a fairly smooth transition from southern to northern Europe, and temperature-related variables are therefore likely to be the underlying variables driving the abundance distribution on a continental scale. Nevertheless, potentially more detailed landscape metrics (such as patch size and edge analysis) may improve future models.

For the Obsoletus and Pulicaris ensembles, our models were able to distinguish between different regions in Europe such as Spain, Germany or Scandinavian countries based on mainly climatic variables. The models performed poorly; however, when predicting the variation in abundance within regions of a country, and especially at a farm level, where the climate is identical but variation in abundance is driven by non-climatic variables. In the case of *C. imicola*, its distribution is confined to southern Europe and the RF approach failed at predicting abundance for this species. It is important to note that all vector data used here are from farms, and therefore are derived from an inherently limited range of land cover. If vector data had been collected at random points land cover variables might have had a much larger effect in the RF models.

Using the same entomological dataset and the same model approach (RF), a previous study showed favourable results in predicting the geographical variation in the probability of presence of these vectors [28]. While most of the literature in the field involve predicting the probability of vector presence, the present study aims to expand the process further and predict vector abundance. We found that vector abundance is more difficult to predict than the probability of presence. This is likely because local abundance depends on factors acting locally e.g. dispersal capabilities, biotic interactions, microenvironment suitability and stochastic effects [46]. Additionally, local abundance usually shows a large variation among nearby locations making the abundance predictions using species distribution models more difficult. Several discrepancies between the predicted probability of presence and site-level abundance have previously been reported in studies of butterflies and vertebrates [47].

Only looking at models with nRMSE values ≤ 2, we found that the most important predictors for the Obsoletes ensemble varied throughout the year, but were all related to temperature (dLST and nLST) and precipitation (BIO 18). Obsoletus ensemble species have a Palaearctic distribution and are widely distributed in central and northern Europe, with low abundance or complete absence in central and southern Spain. The Obsoletus ensemble distribution coincides with humid oceanic climates, characterised by warm summers and a temperate and humid continental climate [48]. Species of the Obsoletus ensemble have also been reported to prefer colder environments where rainfall is regular throughout the year [12]. Our model identified areas with the highest abundance of the Obsoletus ensemble in Germany, followed by France. Versteirt et al. [20] presented an abundance map for *C. obsoletus/C. scoticus* in Europe made by Balenghien & Wint [20]. The spatial pattern shown in our maps is relatively similar, although differences appear in their findings of the highest abundance in western France, in contrast to our results of high abundance in Germany. It is important to take into consideration that the maps presented by Balenghien & Wint [20] were made using two species from the Obsoletus group, while we used species from the Obsoletus ensemble to model and map the spatial abundance. The differences found between our results and theirs might result from the differently grouped species that were used to produce the maps. Another abundance map made by Withenshaw et al. presented in Versteirt et al. [20], showed higher abundance of the Obsoletus ensemble at higher latitudes and decreasing abundance as latitude decreased, similar to our maps.

For the Pulicaris ensemble, the most important variables in the months where the models performed fairly well were related to temperature (BIO 1, BIO 2, nLST and dLST). As with the Obsoletus ensemble, the Pulicaris ensemble has been found in cool and wet climates (with a minimum annual mean temperature of 7 °C and up to 700 mm of rainfall). Our maps showed that the Pulicaris ensemble was widely distributed in Europe, with the highest abundance occurring in northern Germany where abundance was reported to be extremely high in some locations [49], and with high abundance in Poland, in accordance with other studies [27].

The RF models for *C. imicola* had the lowest performance of all the models. The models were not able to predict the highest range of observed abundance, making relatively similar predictions throughout the range of the observed abundance. Nevertheless, our resulting maps displayed a regional *C. imicola* abundance similar to previous studies that modelled *C. imicola* abundance in Spain [15]. Our models were able to recognise environmental factors on a regional scale, which allowed us to estimate the abundance distribution of *C. imicola* quite accurately, as our maps are comparable to those presented in other studies in Spain [14, 24, 50].

The models did not identify variables with a large local variation and therefore could not predict the observed variation in local abundance. Instead, the most important variables identified by our *C. imicola* models were related to temperature (BIO 1 and nLST) and precipitation (BIO 14). Annual mean temperature has been reported to be the main driver of *C. imicola* in Europe. This species is present where temperatures are high on average and stable throughout the year [51, 52]. Precipitation has also been known to affect *C. imicola*, as the species mostly occur where annual rainfall is below 700 mm. [50, 52, 53].

Soil types that are able to retain water, creating muddy, vector-breeding habitats are likely to be of particular importance in the dry Mediterranean climate during the summer months, and soil type variables have been reported to be one of the drivers of *C. imicola* distribution in Spain [16]. In the present analysis, soil type did not appear among the ten most important predictors. One possible explanation could be that the spatial resolution for soil type of 1 km^2^ used here was too coarse to capture the effect on *C. imicola* abundance and that local soil conditions drive abundance of this vector. It could also be that other included variables are correlated with soil type and these variables replaces ‘soil type’ in our models.

## Conclusions

Our RF models were able to distinguish between different regions within nine European countries in terms of average *Culicoides* abundance but resulted in poor predictions of the relatively large observed variation in abundance at the farm level. This may have been due to model limitations, predictor resolution, or lack of important predictor variables. Due to the large amount of trap data used, we were able predict *Culicoides* abundance at the farm level using a simple interpolation approach with nearly the same precision on average as when using an advanced environmental-predictor-driven modelling approach. Model predictions were fair for the Obsoletus ensemble, indicating that our maps could be used as input for more general modelling approaches, such as regional R_0_ models in a monthly resolution for *Culicoides*-borne disease risk assessment. However, there is a need to identify and map the key environmental variables that drive the large variation in abundance we observed between farms in the same region.

## Supplementary information

**Additional file 1: Figure S1.** Yearly variation of the mean abundance for each country. The abundance (y axis) was calculated as the mean all the observations (log transformed) from each country. **Figure S2.** QQ-plots of the residuals per month for the Obsoletus ensemble. **Figure S3**. QQ-plots of the residuals per month for the Pulicaris ensemble. **Figure S4.** Comparison of the abundance maps for each month using Random Forest (RF) and Interpolations for the Obsoletus ensemble. **a** Maps from January to June. **b** maps from July to December. **Figure S5.** Comparison of the abundance maps for each month using Random Forest (RF) and Interpolations for the Pulicaris ensemble. **a** Maps from January to June. **b** Maps from July to December. **Figure S6.** Comparison of the abundance maps for each month using Random Forest (RF) and Interpolations for *Culicoides imicola*. **a** Maps from January to June. **b** Maps from July to December. **Figure S7.** At a local scale, interpolation maps produce a smother surface between the farms compared to environmental driven RF, for which the predictions differ between adjacent pixels. The example shown in the figure corresponds to the August maps for the Obsoletus ensemble. Green dots: farms used for training, purple dots: farms within the test set.

## Data Availability

The national surveillance and research data that support the findings of this study are available from the following people: Spain, Miguel Ángel Miranda Chueca; France, Thomas Balenghien; Germany, Jörn Gethmann; Denmark, Rene Bødker; Sweden, Anders Lindström; Norway, Petter Hopp; Poland, Magdalena Larska; Austria, Katharina Brugger; Switzerland, Alexander Mathis. Restrictions apply to the availability of these data, which were used under license for the current study and are not publicly available. Data are, however, available from the authors upon reasonable request and with permission from the national surveillance programmes of each country.

## Abbreviations

BT: bluetongue disease
RF: Random Forests
MIR: mid-infrared
dLST: daytime land surface temperature
nLST: nighttime land surface temperature
EVI: enhanced vegetation index
NDVI: normalized difference vegetation index
TFA: temporal Fourier analysis
CLC: Corine Land Cover
FAO: Food and Agricultural Organization of the United Nations
nRMSE: normalized root mean square error
