# Supplementary material for: Modelling the monthly abundance of Culicoides biting midges in nine European countries using Random Forests machine learning
Source: Parasit Vectors. 2020 Apr 15;13:194. doi: 10.1186/s13071-020-04053-x (PMC7161244; doi:10.1186/s13071-020-04053-x)
Supplement: Supplementary file 1 — Additional file 1: Figure S1. Yearly variation of the mean abundance for each country. The abundance (y axis) was calculated as the mean all the observations (log transformed) from each country. Figure S2. QQ-plots of the residuals per month for the Obsoletus ensemble. Figure S3. QQ-plots of the residuals per month for the Pulicaris ensemble. Figure S4. Comparison of the abundance maps for each month using Random Forest (RF) and Interpolations for the Obsoletus ensemble. a Maps from January to June. b maps from July to December. Figure S5. Comparison of the abundance maps for each month using Random Forest (RF) and Interpolations for the Pulicaris ensemble. a Maps from January to June. b Maps from July to December. Figure S6. Comparison of the abundance maps for each month using Random Forest (RF) and Interpolations for Culicoides imicola. a Maps from January to June. b Maps from July to December. Figure S7. At a local scale, interpolation maps produce a smother surface between the farms compared to environmental driven RF, for which the predictions differ between adjacent pixels. The example shown in the figure corresponds to the August maps for the Obsoletus ensemble. Green dots: farms used for training, purple dots: farms within the test set. [file 13071_2020_4053_MOESM1_ESM.docx]

**Modelling the monthly abundance of *Culicoides* biting midges in nine European countries using Random Forests machine learning**

Ana Carolina Cuéllar^1*^, Lene Jung Kjær^1^, Andreas Baum^2^, Anders Stockmarr^2^, Henrik Skovgard^3^, Søren Achim Nielsen^4^, Mats Gunnar Andersson^5^, Anders Lindström^5^, Jan Chirico^5^, Renke Lühken^6^, Sonja Steinke^7^, Ellen Kiel^7^, Jörn Gethmann^8^, Franz J. Conraths^8^, Magdalena Larska^9^, Marcin Smreczak^9^,Anna Orłowska^9^, Inger Hamnes^10^, Ståle Sviland^10^, Petter Hopp^10^, Katharina Brugger^11^, Franz Rubel^11^, Thomas Balenghien^12^, Claire Garros^12^, Ignace Rakotoarivony^12^, Xavier Allène^12^, Jonathan Lhoir^12^, David Chavernac^12^, Jean-Claude Delécolle^13^, Bruno Mathieu^13^, Delphine Delécolle^13^, Marie-Laure Setier-Rio^14^, Roger Venail^14,18^, Bethsabée Scheid^14^, Miguel Ángel Miranda Chueca^15^, Carlos Barceló^15^, Javier Lucientes^16^, Rosa Estrada^16^, Alexander Mathis^17^, Wesley Tack^18^ and Rene Bødker^1^

^1^Division for Diagnostics and Scientific Advice, National Veterinary Institute, Technical University of Denmark (DTU), Lyngby, Denmark ^2^Department of Applied Mathematics and Computer Science, Technical University of Denmark (DTU), Lyngby, Denmark ^3^Department of Agroecology - Entomology and Plant Pathology, Aarhus University, Aarhus, Denmark ^4^Department of Science and Environment, Roskilde University, Roskilde, Denmark ^5^National Veterinary Institute (SVA), Uppsala, Sweden ^6^Bernhard Nocht Institute for Tropical Medicine, WHO Collaborating Centre for Arbovirus and Hemorrhagic Fever Reference and Research National Reference Centre for Tropical Infectious Diseases, Hamburg, Germany ^7^Department of Biology and Environmental Sciences. Carl von Ossietzky University, Oldenburg, Germany ^8^Institute of Epidemiology, Friedrich Loeffler Institute, Greifswald, Germany ^9^Department of Virology, National Veterinary Research Institute, Pulawy, Poland ^10^Norwegian Veterinary Institute, Oslo, Norway 11Institute for Veterinary Public Health, Vetmeduni, Vienna, Austria ^12^CIRAD, UMR ASTRE, F-34398 Montpellier, France ^13^Institute of parasitology and tropical pathology of Strasbourg, EA7292, Université de Strasbourg, Strasbourg, France ^14^EID Méditerranée, Montpellier, France ^15^Laboratory of Zoology, University of the Balearic Islands, Palma, Spain ^16^Department of Animal Pathology, University of Zaragoza, Zaragoza, Spain ^17^Institute of Parasitology, University of Zürich, Zürich, Switzerland, ^18^Avia-GIS NV, Zoersel, Belgium.

* Correspondence: anacarocuellar@gmail.com

**Additional file 1: Figure S1**. Yearly variation of the mean abundance for each country. The abundance (y axis) was calculated as the mean all the observations (log transformed) from each country.


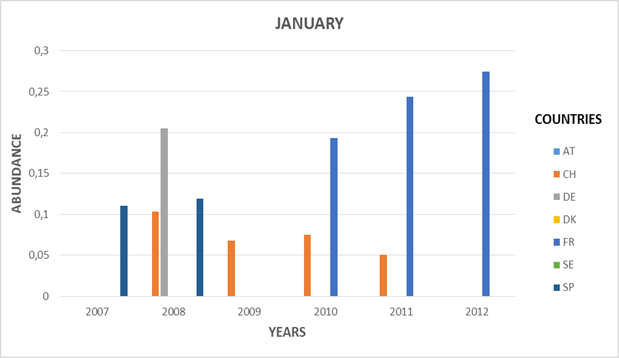


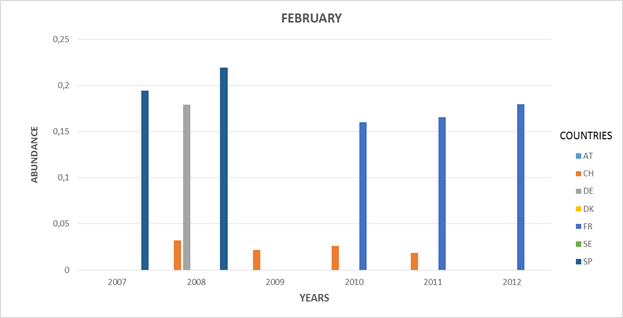


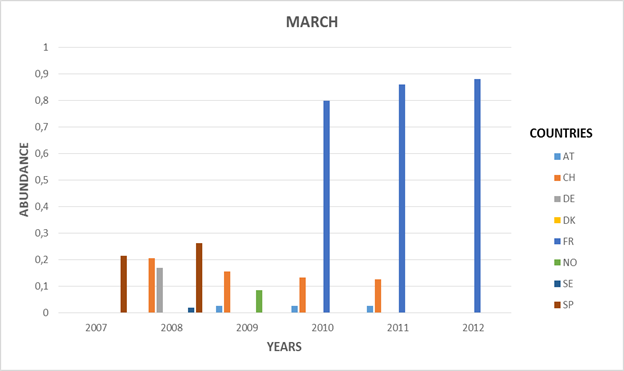


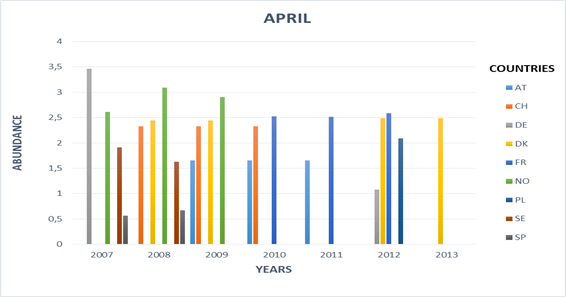


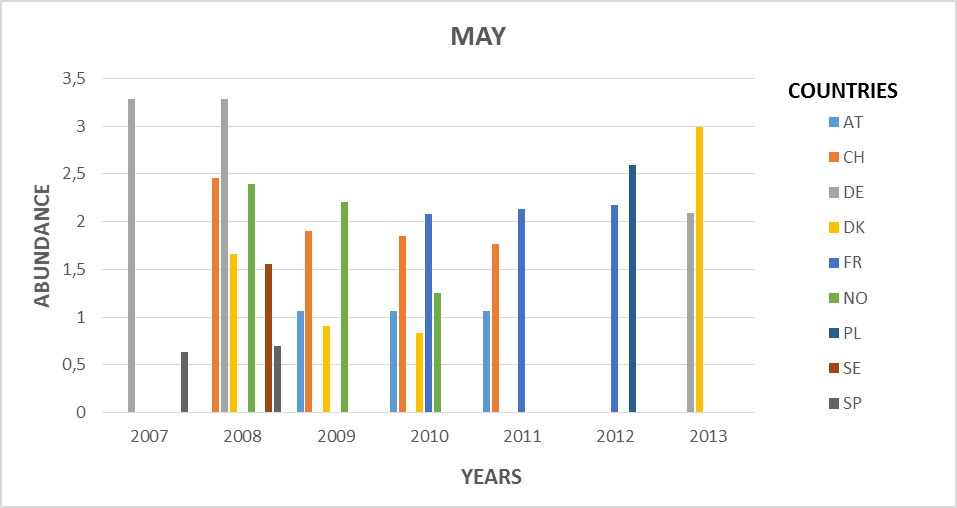


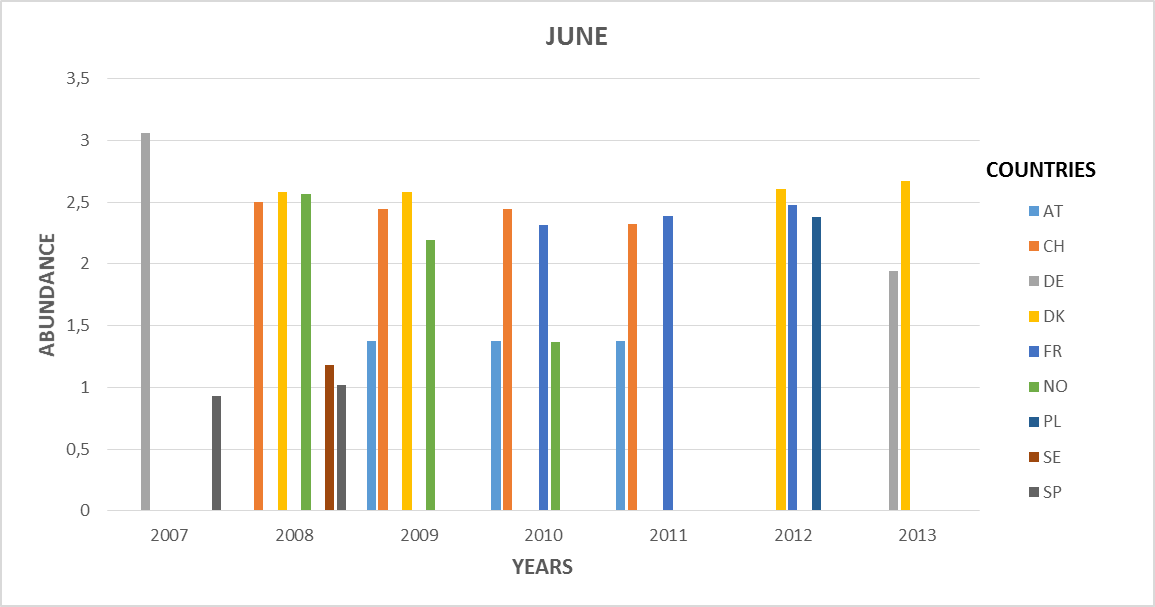


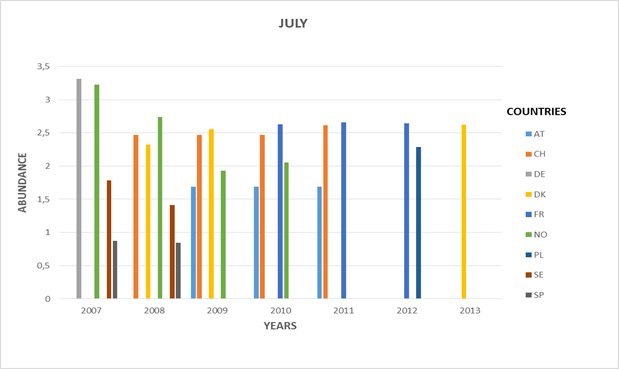


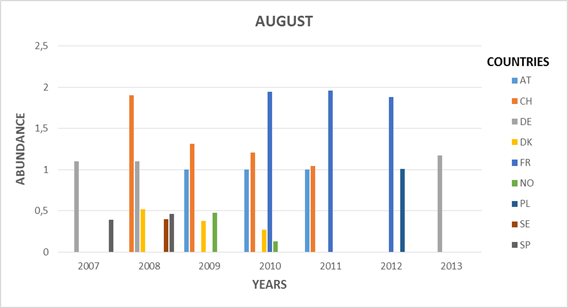


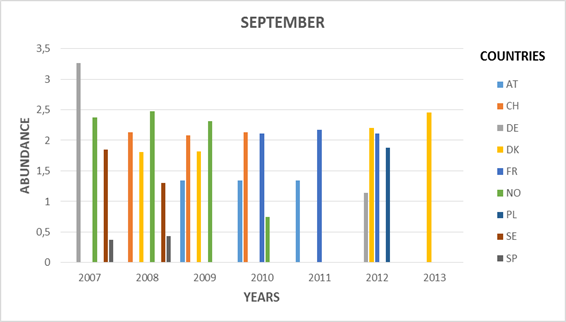


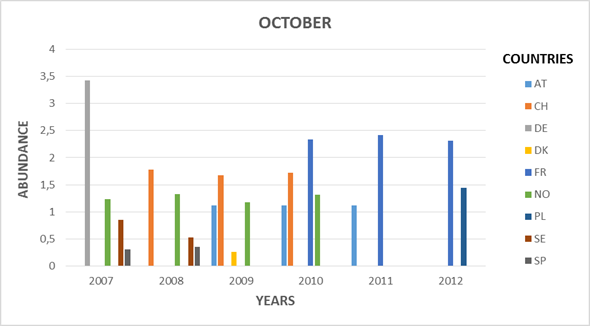


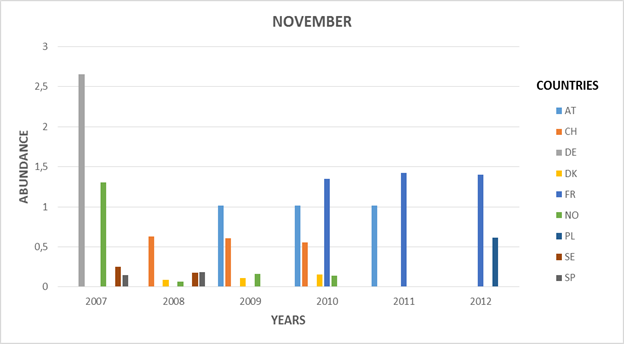


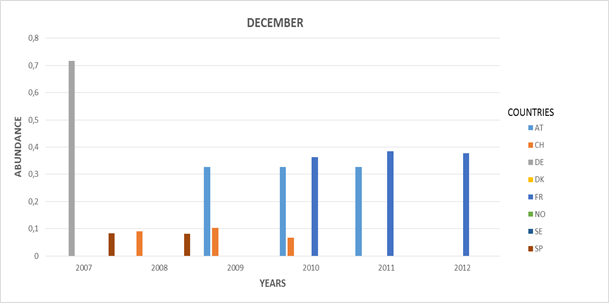


**Additional file 1: Figure S2**. **QQ-plots of the residuals per month for the Obsoletus ensemble.**


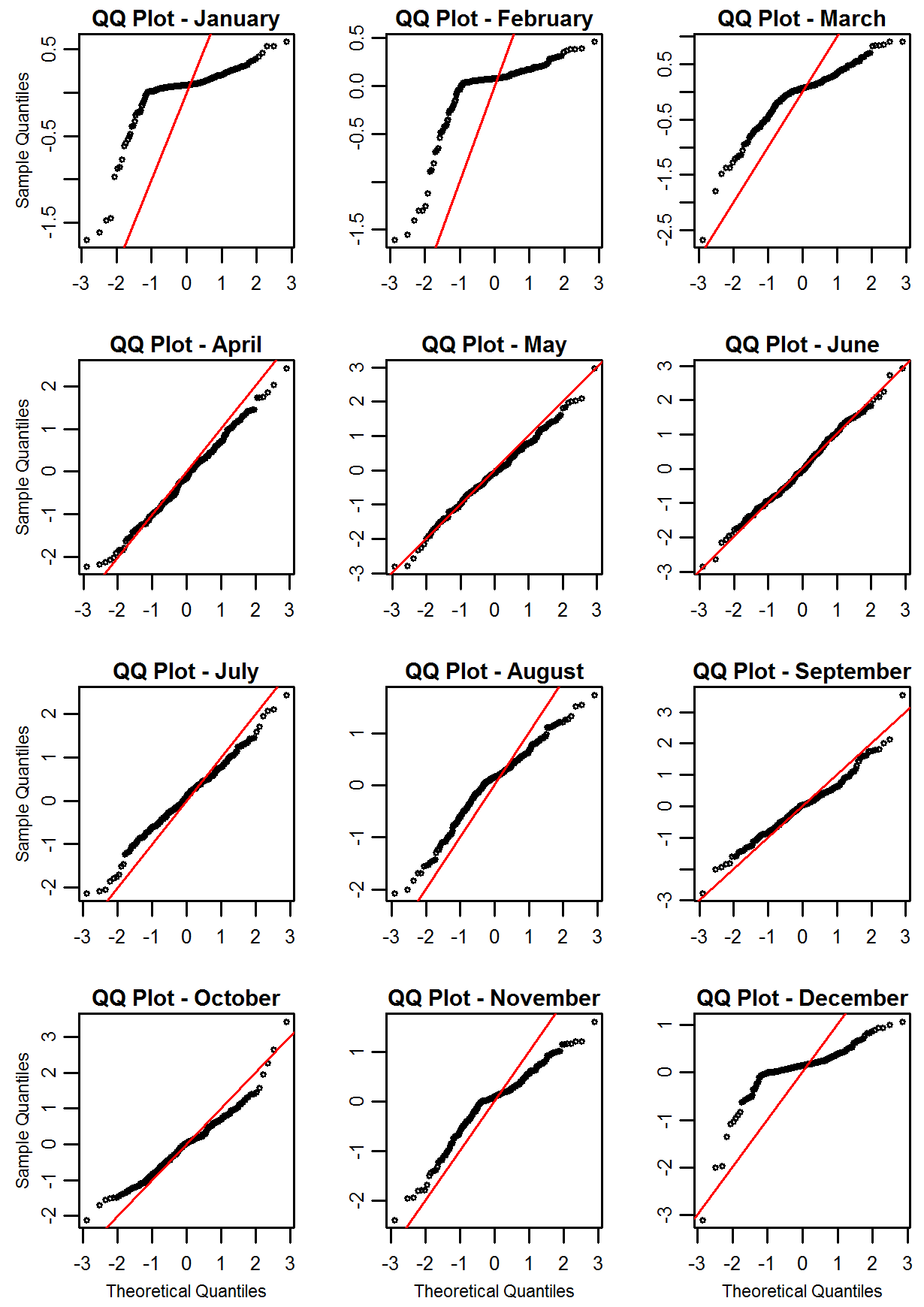


**Additional file 1: Figure S3**. **QQ-plots of the residuals per month for the Pulicaris ensemble.**


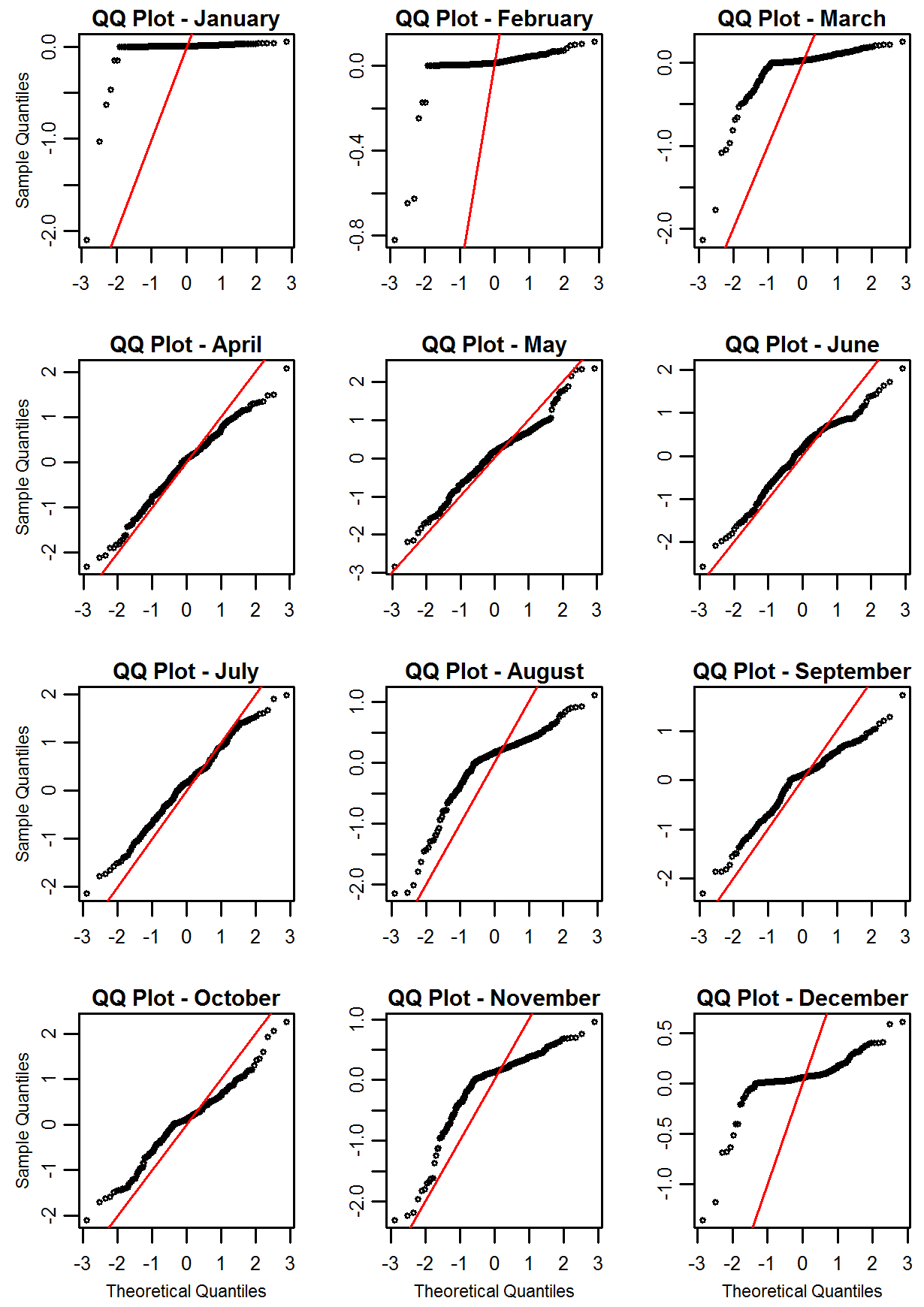


**Additional file 1: Figure S4: Comparison of the abundance maps for each month using Random Forest (RF) and Interpolations for the Obsoletus ensemble. a) maps from January to June. b) maps from July to December.**


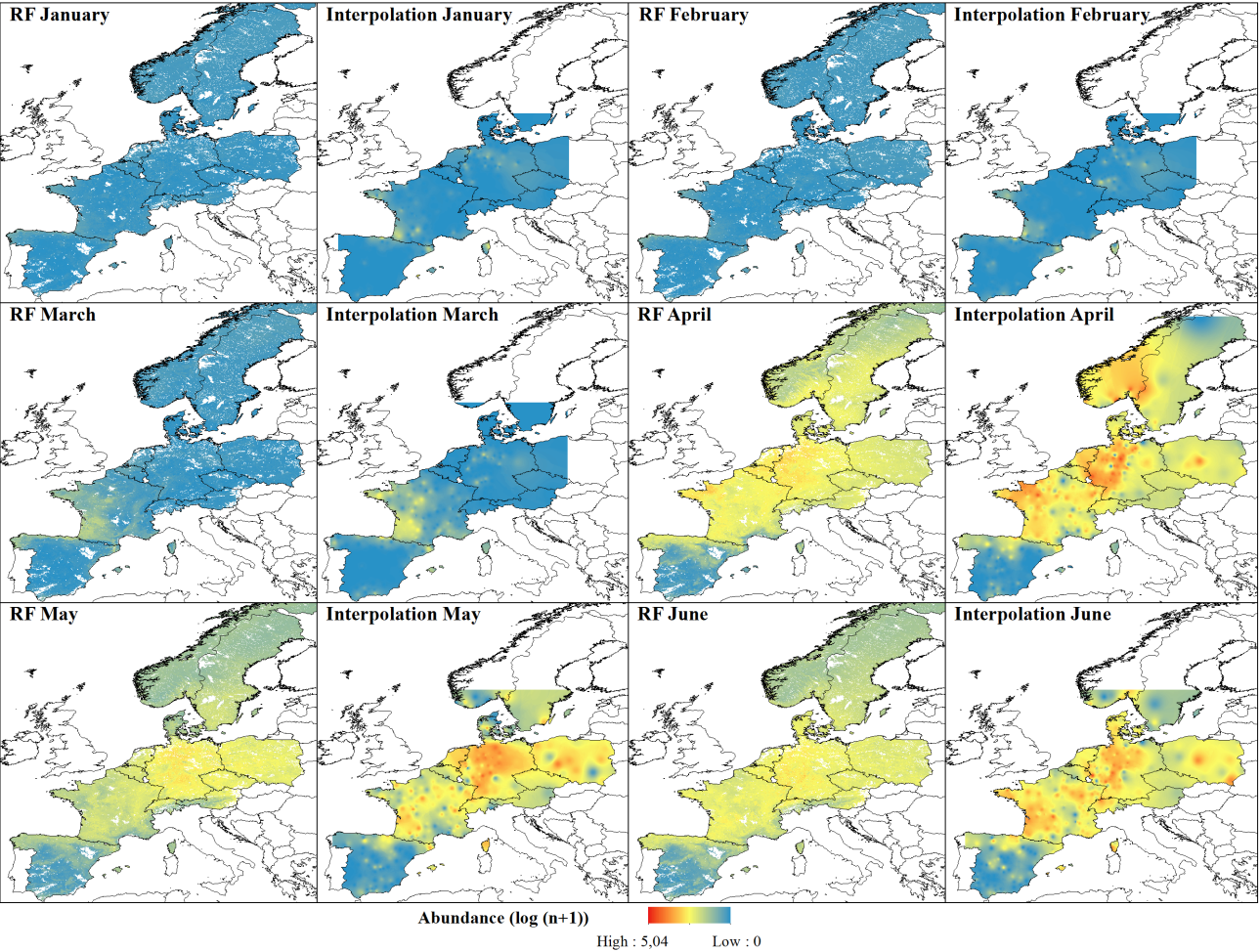


**a**


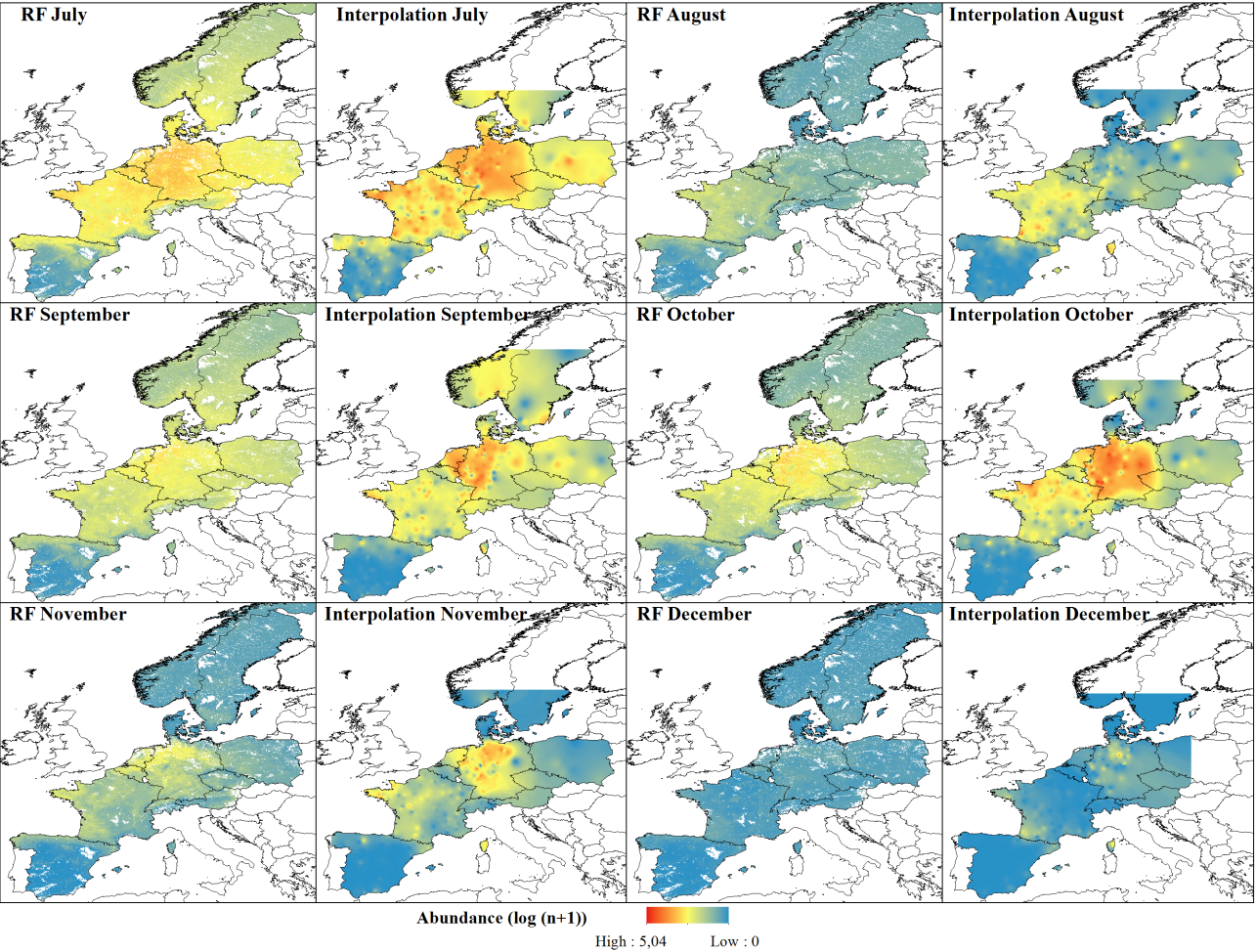


**b**

**Additional file 1: Figure S5. Comparison of the abundance maps for each month using Random Forest (RF) and Interpolations for the Pulicaris ensemble. a) maps from January to June. b) maps from July to December.**


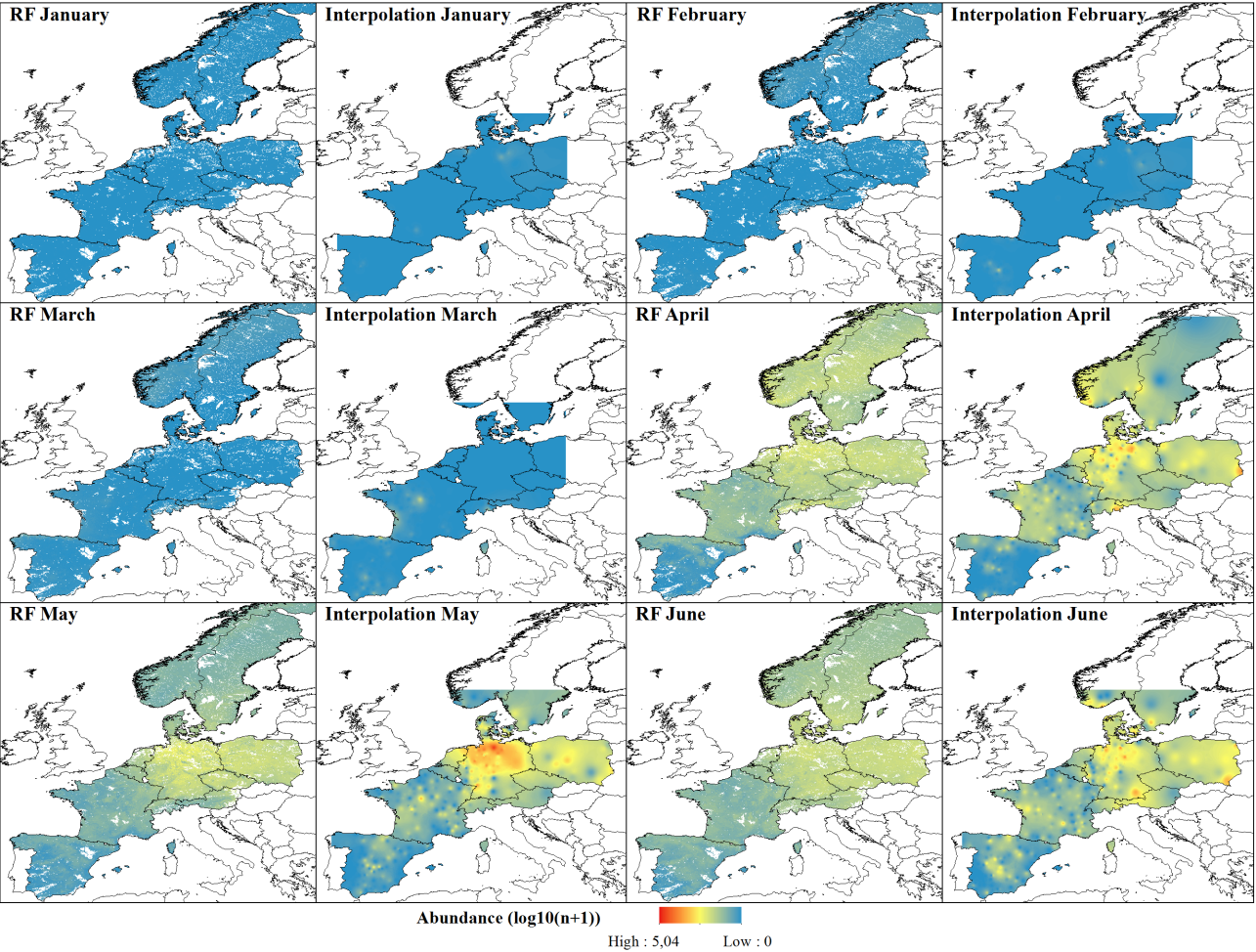


**a**


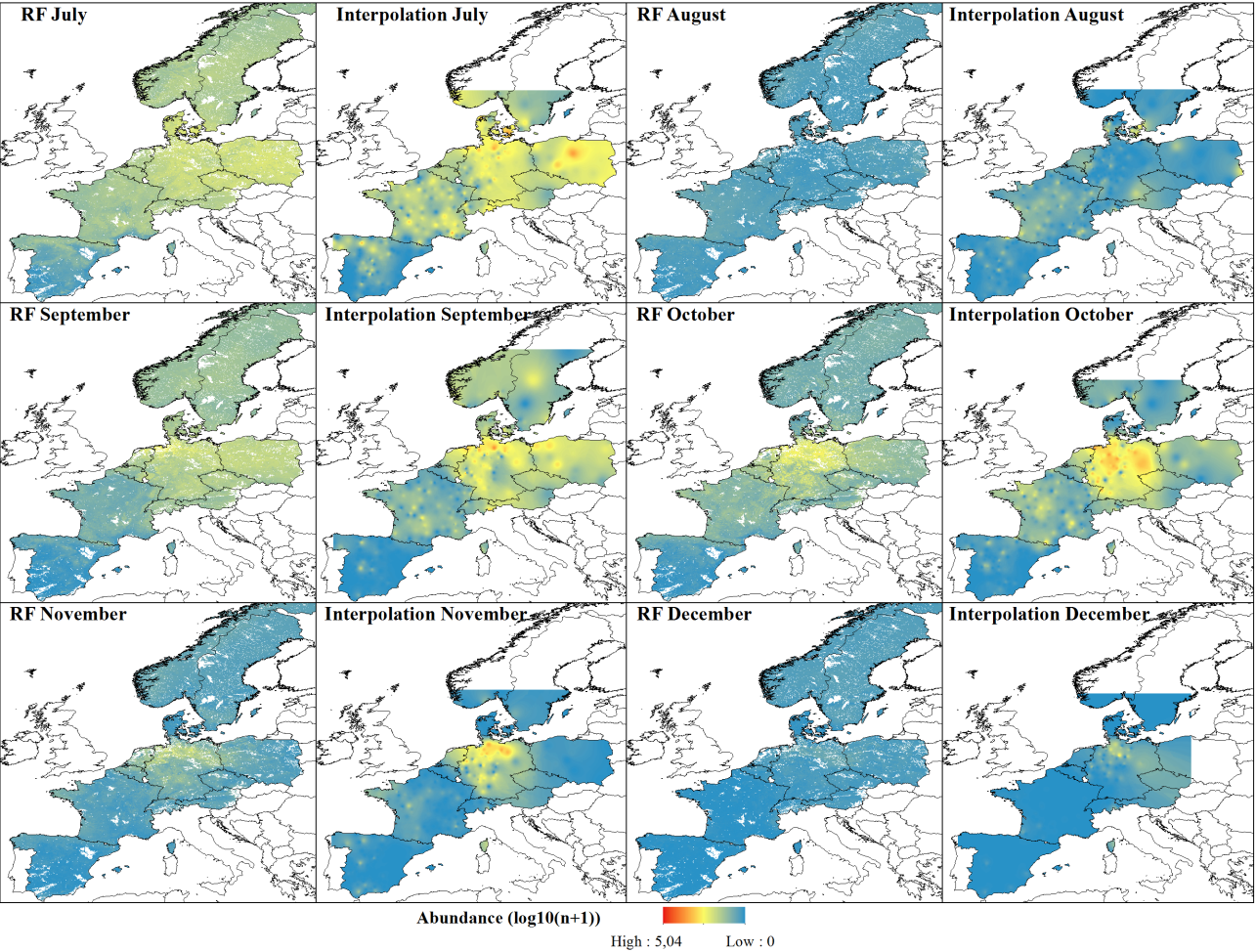


**b**

**Additional file 1: Figure S6. Comparison of the abundance maps for each month using Random Forest (RF) and Interpolations for *Culicoidesimicola*. a) maps from January to June. b) maps from July to December.**


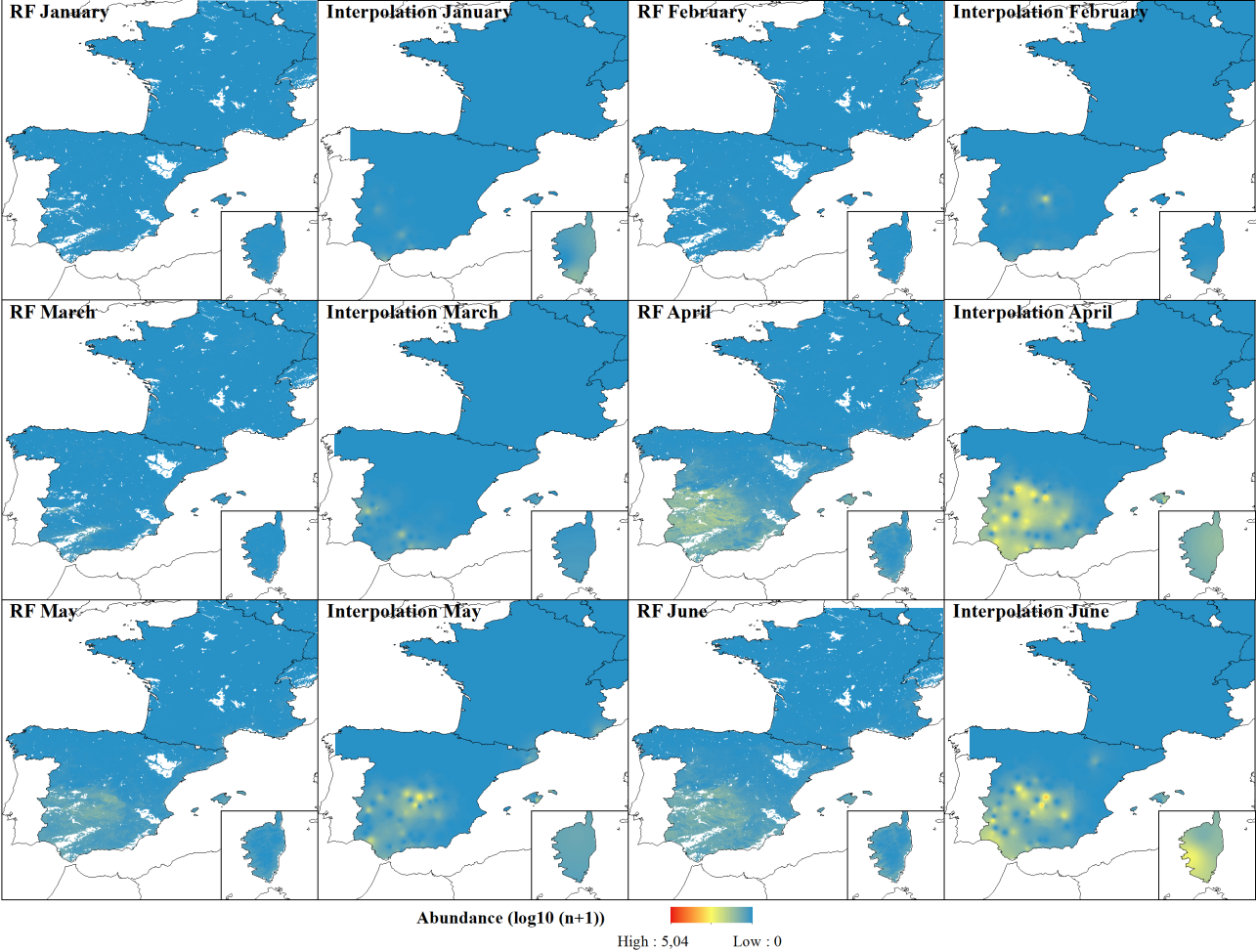


**a**

**b**


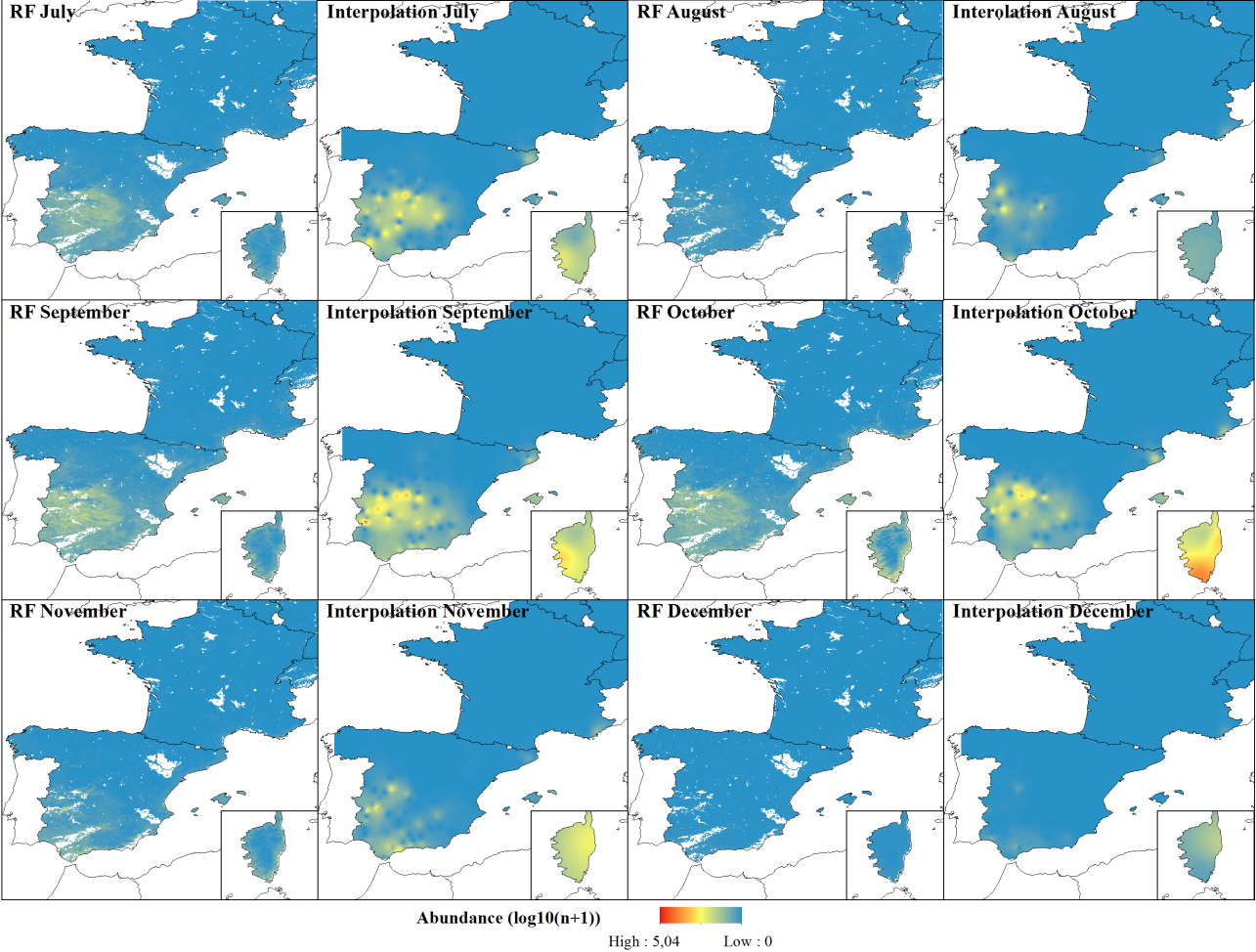


**Additional file 1: Figure S7. At a local scale, interpolation maps produce a smother surface between the farms compared to environmental driven RF, for which the predictions differ between adjacent pixels. The example shown in the figure corresponds to the August maps for the Obsoletus ensemble. Green dots: farms used for training, purple dots: farms within the test set.**


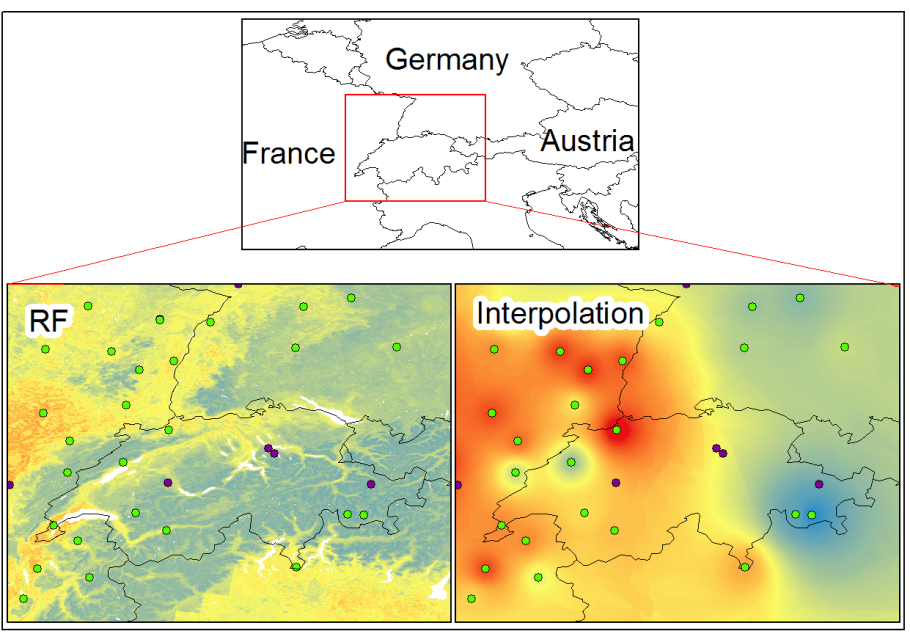


**Additional file 1: Figure S8. Scatterplots showing the observed vs the predicted abundance for the Obsoletus ensemble: a) RF results. b) Interpolation results. Red line: best linear model fit for the predictions. Black line: perfect model fit.**

**
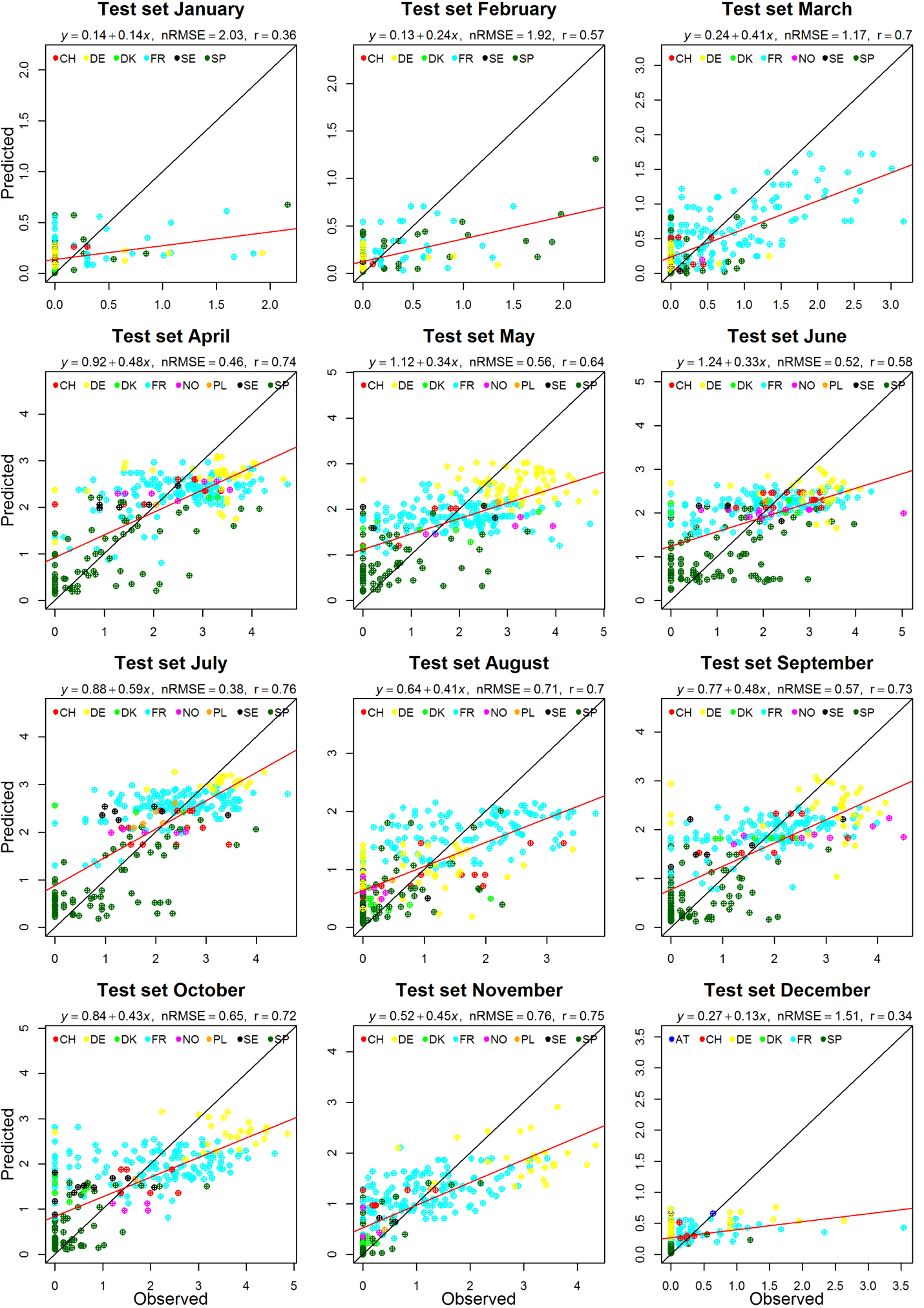
**

1. **Random Forest results**

**
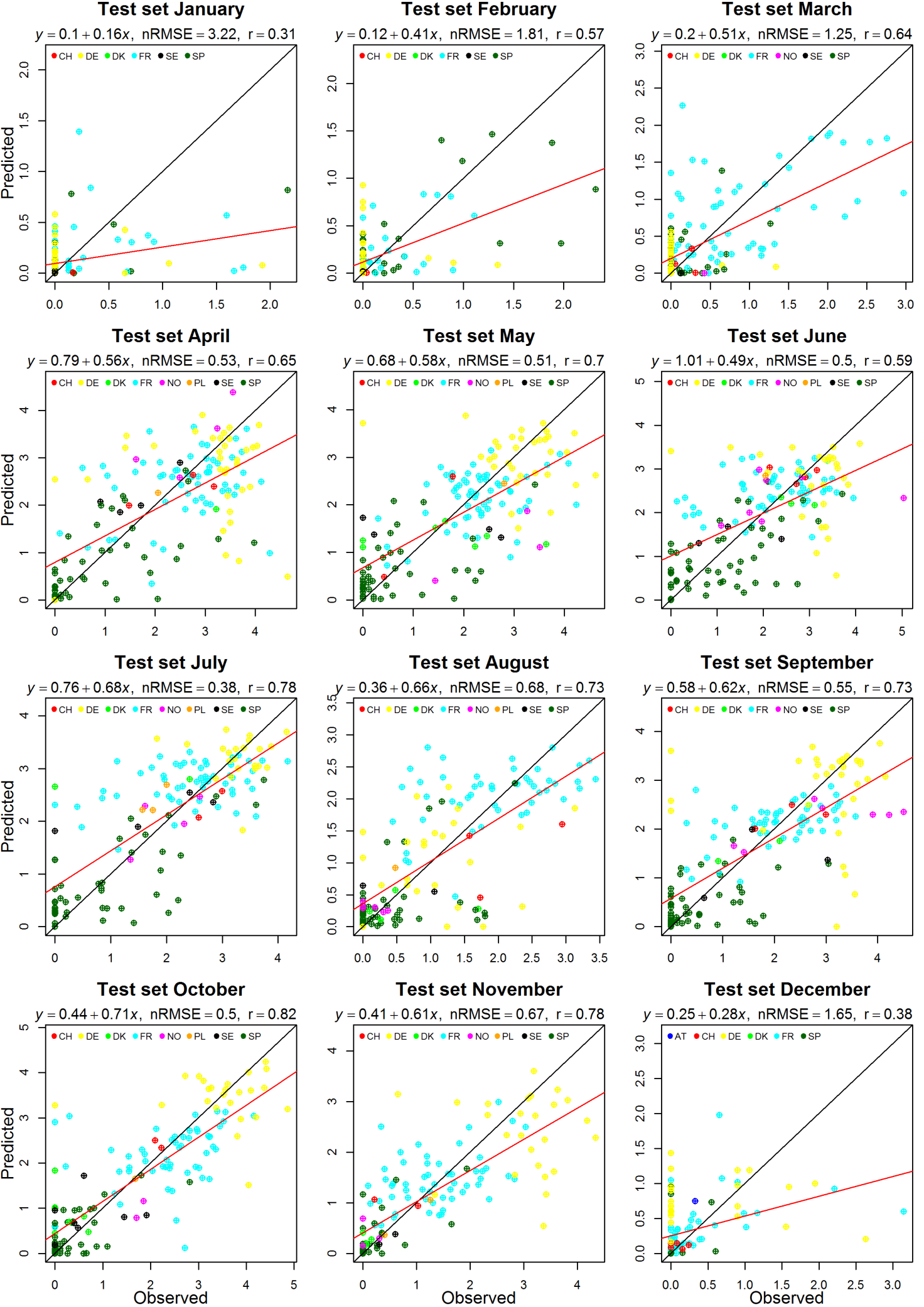
**

1. **Interpolation results**

**Additional file 1: Figure S8. Plotted observed vs predicted values for the Pulicarisensemble: a) RF results. b)Interpolation results. Red line: best linear model fit for the predictions. Black line: perfect model fit.**


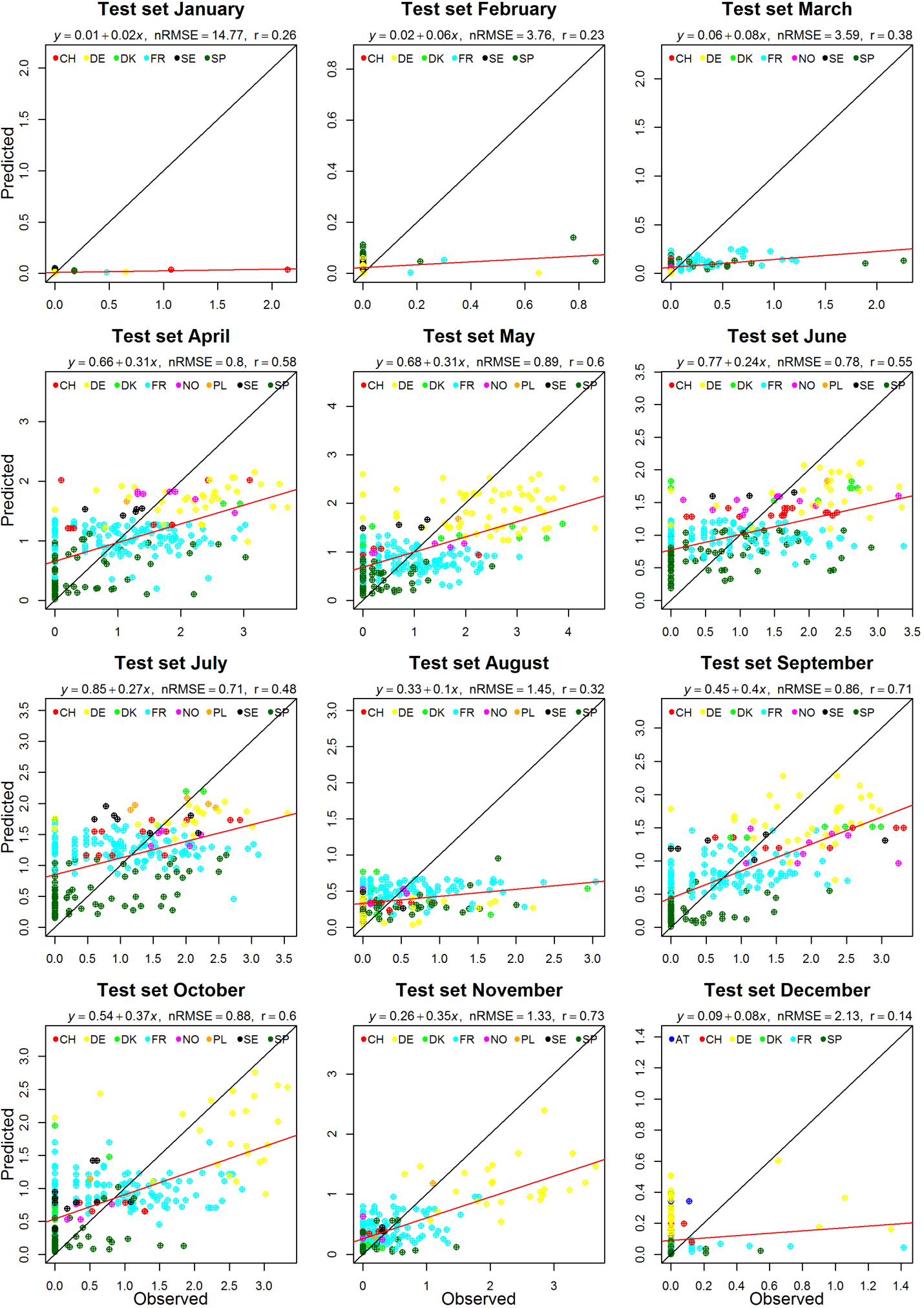


1. **Random Forest results**

**
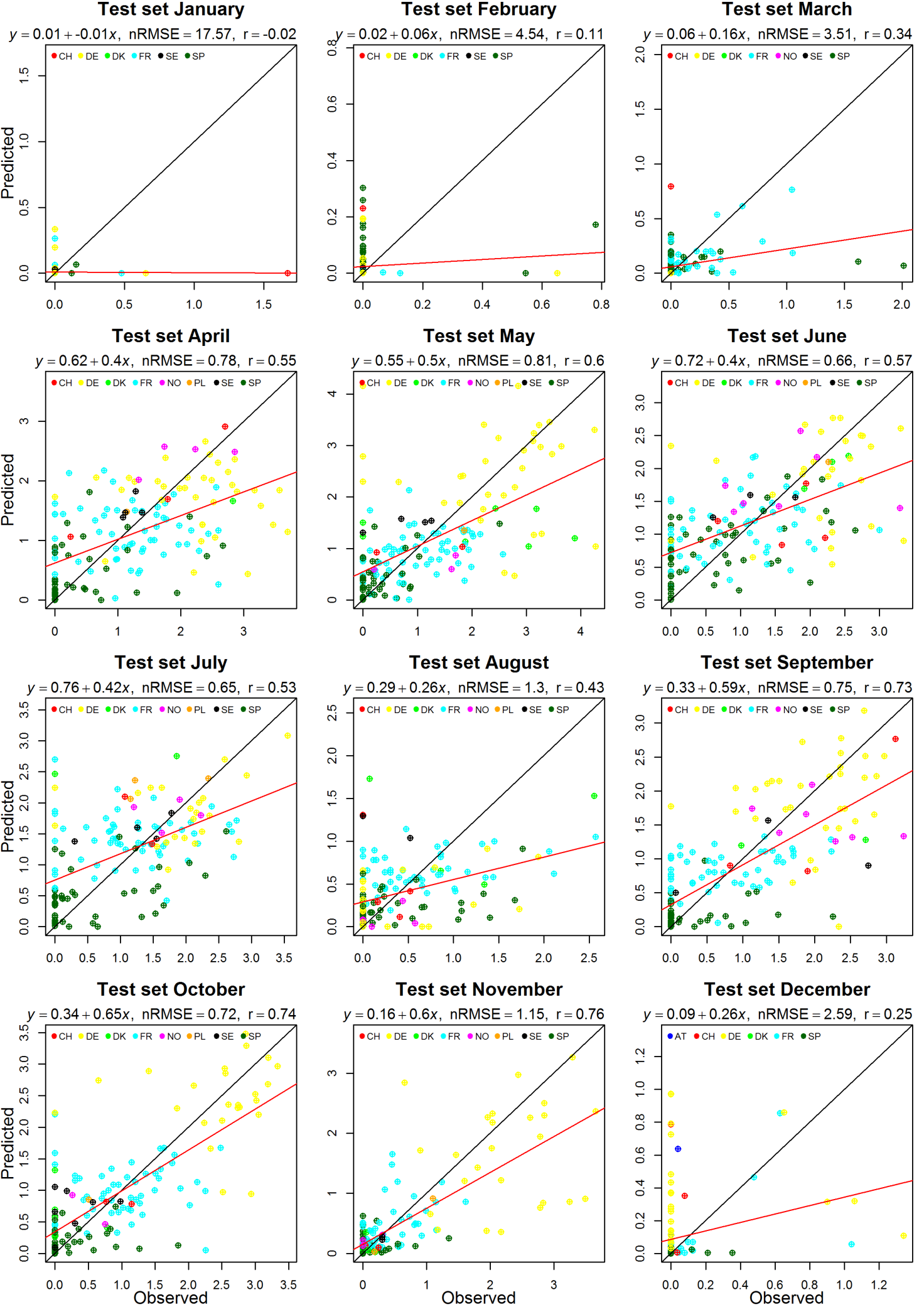
**

1. **Interpolation results**

**Additional file 1: Figure S9. Plotted observed vs predicted values for *Culicoidesimicola*: a) RF results. b) Interpolation results. Red line: best linear model fit for the predictions. Black line: perfect model fit.**


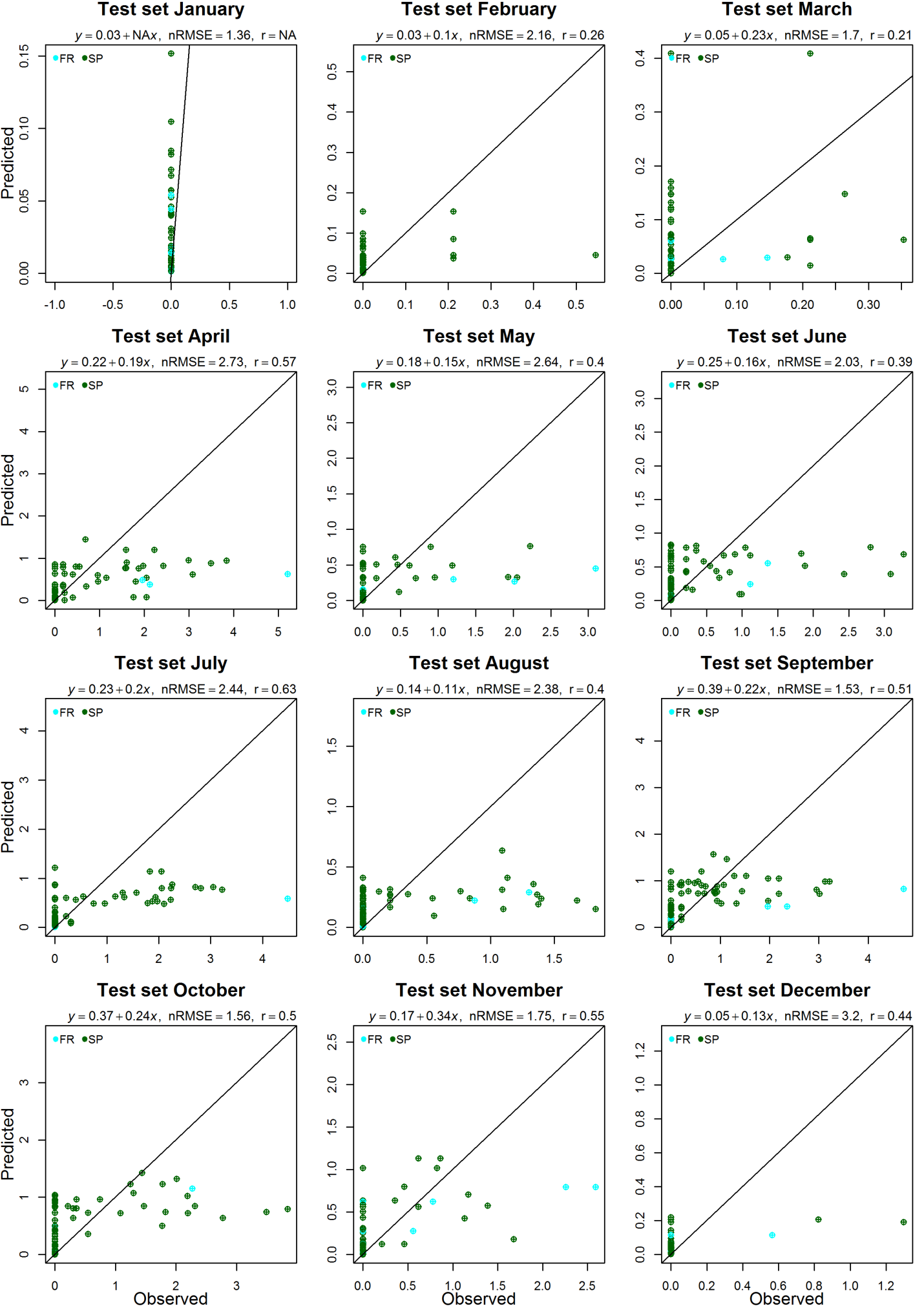


1. **Random Forest results**


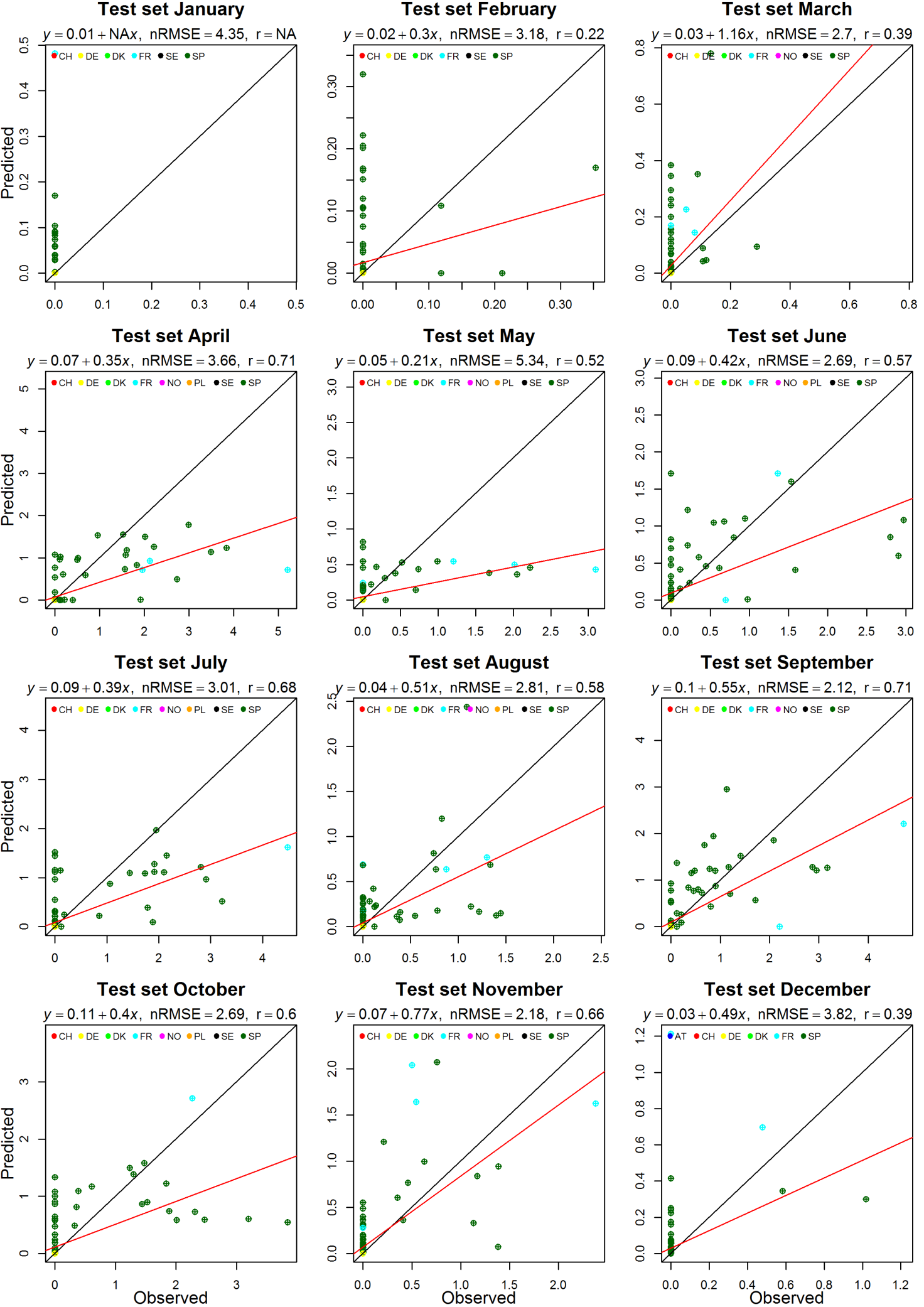


1. **Interpolation results**
